# Highly scalable maximum likelihood and conjugate Bayesian inference for ERGMs on graph sets with equivalent vertices

**DOI:** 10.1371/journal.pone.0273039

**Published:** 2022-08-26

**Authors:** Fan Yin, Carter T. Butts

**Affiliations:** 1 Department of Statistics, University of California at Irvine, Irvine, CA, United States of America; 2 Department of Sociology, Statistics, Computer Science, and EECS and Institute for Mathematical Behavioral Sciences, University of California at Irvine, Irvine, CA, United States of America; Inria - ICM, Paris, FRANCE

## Abstract

The exponential family random graph modeling (ERGM) framework provides a highly flexible approach for the statistical analysis of networks (i.e., graphs). As ERGMs with dyadic dependence involve normalizing factors that are extremely costly to compute, practical strategies for ERGMs inference generally employ a variety of approximations or other workarounds. Markov Chain Monte Carlo maximum likelihood (MCMC MLE) provides a powerful tool to approximate the maximum likelihood estimator (MLE) of ERGM parameters, and is generally feasible for typical models on single networks with as many as a few thousand nodes. MCMC-based algorithms for Bayesian analysis are more expensive, and high-quality answers are challenging to obtain on large graphs. For both strategies, extension to the pooled case—in which we observe multiple networks from a common generative process—adds further computational cost, with both time and memory scaling linearly in the number of graphs. This becomes prohibitive for large networks, or cases in which large numbers of graph observations are available. Here, we exploit some basic properties of the discrete exponential families to develop an approach for ERGM inference in the pooled case that (where applicable) allows an arbitrarily large number of graph observations to be fit at no additional computational cost beyond preprocessing the data itself. Moreover, a variant of our approach can also be used to perform Bayesian inference under conjugate priors, again with no additional computational cost in the estimation phase. The latter can be employed either for single graph observations, or for observations from graph sets. As we show, the conjugate prior is easily specified, and is well-suited to applications such as regularization. Simulation studies show that the pooled method leads to estimates with good frequentist properties, and posterior estimates under the conjugate prior are well-behaved. We demonstrate the usefulness of our approach with applications to pooled analysis of brain functional connectivity networks and to replicated x-ray crystal structures of hen egg-white lysozyme.

## 1 Introduction

Networks are relational structures composed of individual entities (*vertices* or *nodes*) together with a set of pairs or ordered pairs of entities (*ties* or *edges*) that share a specific relationship. Networks arise in many scientific fields, ranging from biology and epidemiology to social science and engineering. For example, social science researchers are frequently interested in interpersonal networks, in which nodes correspond to individuals and edges represent personal relationships (e.g., friendship [1], advice-seeking [2], etc.); in biology, there has been research interest in using networks to represent complex phenomena such as transcriptional regulation [3], trophic systems [4], interspecific competition [5], animal social interaction [6], protein structure [7] and aggregation [8], and the structure and function of neural systems [9]. As these disparate examples illustrate, networks have proven to be a fruitful framework for treating a wide range of phenomena, and research on network structure has grown apace.

Accompanying this growth has been a corresponding literature on techniques for network measurement, modeling, and analysis. Recent advances in inferential methods for models with complex dependencies have enabled the statistical modeling and analysis of network data to become a practical tool for a growing range of research applications [10–12]. A variety of modeling approaches have benefited from these advances, particularly exponential family random graph models (ERGMs) [13], parametric model families that are capable of capturing the complex dependence structure that is typical of network data. ERGMs (known in older work as p* models, e.g., [14]) gradually developed from early tools for testing dependence hypotheses to a general framework for modeling networks with heterogeneity and complex dependence [15–19], and have spawned a growing body of theoretical [20–27] and methodological work [28–34]. (See [35] for a recent review.) ERGMs have been widely applied in many scientific fields, for example, including (but not limited to) sociology [1, 36, 37], political science [38], bioinformatics [3], public health [39], biophysics [8, 40], and neuroscience [41, 42].

Research on statistical network models has been particularly concerned with the case of inference from a single network observation, but multiple random network realizations are also increasingly common in practice, and can be divided into major two categories depending upon the nature of the underlying vertex set. The first category involves cases such as networks from independently solved protein structures or molecular dynamics (MD) simulations, brain functional connectivity networks [41–43], or dynamic friendship networks within fixed groups [44], where the node sets on which the relationship is defined are constant across observations. There are also cases where node sets across different observations are potentially non-equivalent, including comparative studies of networks across groups [45] or species [46], intervention studies for education research [47, 48] and friendship network studies across different schools [36], or studies of emergent multi-organizational networks with changing composition [49]. A second useful distinction involves cases in which multiple independent (or approximately independent) networks are observed (e.g., from populations of subjects or organizations, or from well-spaced snapshots of equilibrium dynamics), versus time series data in which draws are strongly autocorrelated. The latter is heavily studied as a case in its own right (e.g., [50–55]), with a primary focus on uncovering the mechanisms governing network dynamics. Models for the former case generally seek to either strictly summarize structural variation in a population of graphs (e.g., [46, 56]) or pool information in a common population model (e.g., [45, 47, 48, 57]) that infers common structural tendencies in a more generative fashion. As we detail below, our focus in this paper is on models for the equivalent vertex set/non-autocorrelated pooled case.

Despite considerable progress in this area, there remains room for improvement in approaches for inference based on multiple network observations with ERGMs. Broadly speaking, existing approaches based on full likelihood calculations (as opposed to composite/pseudo-likelihood methods, e.g., [38, 40, 46]) can be divided into two categories, unpooled and pooled estimates. Unpooled estimation employs an essentially meta-analytic approach, in which a proposed model is fit to each network observation separately, with the resulting parameter estimates being jointly analyzed in a second stage. For example, motivated by the goal of finding a model for brain-connectivity networks at the group level [42], specified an ERGM model for brain-connectivity networks and then fit the model to each individual separately, subsequently combining the resulting estimates by taking their respective means and medians as point estimates of the parameters of a group-level representative model. A similar approach was used by [36] in studying friendship across schools, with separate models fit to each network and the resulting statistics then summarized to infer general patterns. Under such a framework, the time complexity of inference scales linearly in the number of graphs, making this generalization expensive as the number of networks grows (particularly if the individual networks are themselves large). A second problem with the unpooled approach is that it may be difficult or impossible to find a model that is both estimable on each individual network and that includes all effects of substantive interest. For instance, where the model sufficient statistics for a particular network are sufficiently extreme (in a sense to be clarified below), the MLE may not exist; this condition is common for effects involving subgroup interactions when said subgroups are small. Importantly, this failure need not mean that the model family is generally inappropriate or ill-behaved, instead stemming from limitations in the ability to fit some models to a single graph realization. A natural way to avoid such problems is by pooled estimation. In the ERGM context, pooled estimation has been studied in cases such as independently observed intraschool friendship networks [58], where the adjacency matrices representing the observed networks of each distinct school are aggregated to a block-diagonal matrix with structural zeros assumed for all off-diagonal blocks. However, the high cost of performing MCMC MLE on such a pooled network greatly limits the scale of cases that can be considered in this way. Similar schemes using hierarchical Bayesian models have also been proposed [59], but require high-quality MCMC simulations that can be computationally demanding when the number of network observations is large. Importantly, all of these methods share the property that likelihood calculations must *de facto* be performed for each network separately (whether those networks are notionally joined together in one large synthetic network as part of a pooling scheme or treated separately), which greatly increases both storage and time complexity (especially for large graphs).

This cost poses a substantial barrier in applications such as neuroscience or biophysics, where pooled inference on large collections of networks is of potential interest. Importantly, however, some of these cases have the special property that the collections of networks to be analyzed (or large subsets thereof) involve equivalent vertex sets (with either absent or equivalent covariates). For instance, [40] study two collections of approximately 1,000 networks representing independently drawn local energy minima for structures of wild type and E22G variants of A*β*_1–40_, a protein that plays a key role in the etiology of Alzheimer’s disease. Each collection represents a series of independent draws from a respective common graph distribution, with identical vertex properties for graphs in each set. Likewise, in neuroscience settings one may (as in e.g., [41]) observe anatomically defined networks on collections of subjects that are (at least provisionally) considered exchangeable within groups, and that can be usefully modeled as draws from a common graph generating process. Although less common to date in social science settings, collections of exchangeable networks with equivalent vertex sets can arise for example from behavior in human subject experiments [60], semantic networks extracted experimentally or from texts [61], or as the result of replicated outcomes of agent-based simulations [62]. In such cases, it is possible to perform pooled ERGM inference at vastly lower cost than is possible with conventional techniques—indeed, obtaining computational costs for estimation that are identical to the single-graph case.

In this paper, we propose a novel method for fitting multiple graph observations embedded in equivalent node sets using a pooled ERGM approach. By exploiting a simple property of statistical exponential families, we are able to convert the problem of pooled inference for a (possibly large) graph set to the problem of inference for a single pseudo-graph of size equal to an individual input graph, subsequently correcting the single graph information matrix for the sample size of the pooled data set. We also show that a minor adjustment to this technique can be used to perform maximum *a posteriori* (MAP) estimation under conjugate priors at no additional cost, either for a single graph or for multiple graph observations. Because our technique works entirely within the *mean value space* of the chosen ERGM family, it can be performed via data adjustments with existing software intended for single-graph estimation, and is compatible with any estimation method that works via sufficient statistics (including the widely used Geyer-Thompson [63, 64] and stochastic approximation [65] methods). In addition to pooling information and providing inexpensive Bayesian answers (using the Laplace approximation to the posterior distribution), our approach provides a simple and effective mechanism for regularization, with particular virtue in resolving the “convex hull problem” that frequently arises in discrete exponential families (see Section 2.2); we describe a simple approach to prior specification that is well-suited to this purpose, and that can be easily extended to provide more informative priors when appropriate background information is available.

The remainder of this paper is organized as follows. We begin with general concepts and notation for ERGMs in Section 1.1, and present our framework for scalable inference under both frequentist and Bayesian settings (including issues of prior specification) in Section 2. In Section 3, we employ simulation studies to examine the performance of our approach, and the behavior of posterior inference as a function of prior weight. In Section 4, our proposed methods are demonstrated on two different multiple network applications: brain functional connectivity networks from multiple subjects (Section 4.1); and protein structure networks from replicated x-ray crystal structures of hen egg-white lysozyme (Section 4.3). Finally, we close with a brief discussion and comment on potential future work.

### 1.1 Exponential family random graph models

Consider an order-*n* graph, *G*, represented via adjacency matrix *Y* on support $Y_{n}$, such that *Y*_*ij*_ corresponds to the state of the edge between vertices *i* and *j*; we make no particular assumptions about $Y_{n}$ (e.g., it may consist of directed or undirected graphs, with or without loops, and may be valued), save that all elements of $y\in Y_{n}$ are real and finite. An *exponential family random graph model* (ERGM) for *Y* is then given by

$$\begin{array}{c} P_{\eta }\left(Y=y\right)=h\left(y\right)\text{exp}\{\eta {\left(\theta \right)}^{⊺}g\left(y\right)-\psi \left(\eta \left(\theta \right)\right)\} \end{array}$$ (1)

where $g:Y_{n}\to R^{p}$ is a vector of real-valued sufficient statistics capturing network features of interest (which may implicitly incorporate e.g., nodal or dyadic covariates) and $\theta \in \Theta \subseteq R^{q}$ is vector of (curved) model parameters mapped to canonical parameters $\eta :\theta \to R^{p}$ [19]. The reference measure *h* determines the baseline behavior of the ERGM distribution when *η*(*θ*) = 0, and plays an important role in fixing the shape of the distribution when edges are valued [32]. In general, computation involving (1) is challenging due to the intractable nature of the log-partition function (i.e., normalizing factor), $\psi \left(\eta \left(\theta \right)\right)=\text{log}\sum_{y^{\prime }\in Y_{n}}h\left(y^{\prime }\right)\text{exp}\{\eta {\left(\theta \right)}^{⊤}g\left(y^{\prime }\right)\}$, as $|Y_{n}|$ is extremely large ($O(2^{n^{2}})$ in the binary case), the summand is generally too rough for naive Monte Carlo strategies to converge well, and *ψ* rarely has a closed-form solution. In the context of iid draws from the same ERGM pmf, we obtain the (homogeneous) *pooled ERGM*,

$$\begin{array}{c} P_{\eta }\left(Y=y\right)=\text{exp}\{\eta {\left(\theta \right)}^{⊺}\sum_{i=1}^{m}g\left(y^{i}\right)+\sum_{i=1}^{m}\text{log}h\left(y^{i}\right)-m\psi \left(\eta \left(\theta \right)\right)\}, \end{array}$$ (2)

where **Y** = (*Y*^1^, …, *Y*^*m*^) is a vector of random graphs with realizations **y** = (*y*^1^, …, *y*^*m*^). Although most work focuses on the single-graph case, our emphasis here is on the case where *m* > 1 (either because of multiple graph observations, or—in the case of conjugate prior inference—because of “effective” prior observations that are equivalent to an increased *m*).

As exponential families, the ERGMs have a number of convenient properties of which we will make use [35]. Subject to mild regularity conditions, we may define an invertible function $\mu \left(\eta \right)=E_{\eta }g\left(Y\right)$ that provides the *mean value parameterization* of an ERGM on random graph *Y*. From Eq 2 it is evident that the corresponding function $\mu_{m}\left(\eta \right)=E_{\eta }g\left(Y\right)=m\mu \left(\eta \right)$ is simply a constant multiple of the base mean value function for a single graph (foreshadowing a property that we employ below). Likewise, the Fisher information matrix of *Y* is given by $I\left(\eta \right)=E_{\eta }[\nabla \text{ln}P_{\eta }\left(Y\right){\left(\nabla \text{ln}P_{\eta }\left(Y\right)\right)}^{⊤}]={\text{Var}}_{\eta }g\left(Y\right)$, with the pooled equivalent being ***I***_*m*_(*η*) = *m* ***I***(*η*).

## 2 Mean value inference for pooled ERGMs

Although a number of variants exist, standard approaches to inference for pooled ERGMs share the basic approach of computing likelihoods (or in some cases pseudo-likelihoods) for all observed graphs, and using the resulting joint likelihood for inference. Computationally, this may involve (as e.g., in [58]) combining the observed graphs into a single large synthetic network of order |*V*| = *mn* (with support constraints prohibiting cross-graph ties), and then performing MCMC MLE or comparable Bayesian analyses on the synthetic graph; for pseudo-likelihood methods (e.g., [30, 66]), edge variables may simply be combined across networks, possibly with resampling over networks (as with bootstrap [53, 67] or Bayesian bootstrap [40] strategies). These strategies lead to computational and storage costs that increase at least linearly in the number of graphs, which can become prohibitive for large systems or when the number of graphs is substantial. Here, we observe that a much faster strategy based on the mean values of the sufficient statistics becomes available in the IID case, and that this same strategy can also be leveraged for conjugate Bayesian inference. To our knowledge, this very simple but powerful trick has not previously been exploited in the ERGM context.

### 2.1 Maximum likelihood inference for pooled ERGMs

We begin with the simple case of maximum likelihood inference. Given IID ERGM observations ***y***^*obs*^ = (*y*^1^, *y*^2^, ⋯, *y*^*m*^), the joint log-likelihood follows immediately from Eq 2,

$$\begin{array}{c} \ell \left(\theta ;y^{obs}\right)=\eta {\left(\theta \right)}^{⊺}\sum_{i=1}^{m}g\left(y^{i}\right)+\sum_{i=1}^{m}\text{log}h\left(y^{i}\right)-m\psi \left(\eta \left(\theta \right)\right), \end{array}$$ (3)

the maximizer of which ($\hat{\theta }=\text{arg}{\text{max}}_{\theta }\ell \left(\theta ;y^{obs}\right)$) is the maximum likelihood estimator (MLE). As observed, the primary challenge in finding the MLE is in dealing with the log normalizing factor, *ψ*. Running a Markov chain over the states of each of the *m* graphs in the set can be used to accomplish this, or equivalently (as is done in e.g., [58]) running a single Markov chain on a combined graph of order *nm* containing the union of all individual graphs, but the form of Eq 3 shows that this is superfluous in the IID case. Specifically, observe that any maximizer of *ℓ* is also a maximizer of any positive constant multiple of *ℓ*, and thus

$$\begin{array}{cc} \hat{\theta } & =\text{arg}\;\underset{\theta }{\text{max}}\;\ell \left(\theta ;y^{obs}\right)\\ & =\text{arg}\;\underset{\theta }{\text{max}}\;\ell \left(\theta ;y^{obs}\right)/m\\ & =\text{arg}\;\underset{\theta }{\text{max}}\;\eta {\left(\theta \right)}^{⊺}\bar{g}\left(y^{obs}\right)+\text{log}\tilde{h}\left(y^{obs}\right)-\psi \left(\eta \left(\theta \right)\right), \end{array}$$

where $\bar{g}\left(y^{obs}\right)=\frac{1}{m}\sum_{i=1}^{m}g\left(y^{i}\right)$ is the arithmatic mean of the observed statistics, and $\tilde{h}\left(y^{obs}\right)=\text{exp}[\frac{1}{m}\sum_{i=1}^{m}\text{log}h\left(y^{i}\right)]$ is the geometric mean of the reference measure over the observed graphs. Since the latter does not depend on *θ*, we may further simplify the above to

$$\begin{array}{c} \hat{\theta }=\text{arg}\;\underset{\theta }{\text{max}}\;\eta {\left(\theta \right)}^{⊺}\bar{g}\left(y^{obs}\right)-\psi \left(\eta \left(\theta \right)\right), \end{array}$$ (4)

which is immediately recognizable as the MLE for a hypothetical *single* “pseudo-graph” of order *n* with whose statistics are the means of the observed statistics. It is thus possible to find the MLE for a pooled model on *m* graphs by fitting a single-graph model (a considerable simplification).

To see the corresponding implications for the sampling distribution of the MLE, we note that inference for *θ* benefits from standard asymptotics in *m* (see e.g., [35]), including the consistency and asymptotic normality of the MLE under suitable regularity conditions. In particular, if ${\hat{\theta }}_{m}$ is the MLE for **Y** with *m* observations drawn from a pooled ERGM with parameter *θ*_0_, then it follows from standard exponential family theory [68] that

$$\begin{array}{c} \sqrt{m}\left({\hat{\theta }}_{m}-\theta_{0}\right)\overset{D}{\to }N\left(0,I^{-1}\left(\theta \right)\right) \end{array}$$ (5)

where ***I***(*θ*) can be obtained from *I*(*η*) via the chain rule, i.e., $\nabla \eta {\left(\theta \right)}^{⊤}I\left(\eta \right)\nabla \eta \left(\theta \right)$. It thus follows that the asymptotic variance-covariance matrix of the MLE in the *m*-graph case is equal to that of the single-graph case, divided by *m*; i.e.,

$$\begin{array}{c} \text{Var}{\hat{\theta }}_{m}\to \frac{1}{m}\nabla \eta {\left(\theta \right)}^{⊺}[\text{Var}g(Y)]\nabla \eta \left(\theta \right). \end{array}$$ (6)

It follows, then, that we may perform maximum-likelihood inference for ***y***^*obs*^ with arbitrarily large *m* *at no greater cost than fitting to a single network* (and without the use of customized software tools): we simply find the MLE for the a single (imaginary) graph with statistics equal to the mean of the observed statistics using any standard method (e.g., MCMCMLE [30] or stochastic approximation [65]), and then rescale the associated variance-covariance matrix by a factor of *m* to correct for sample size. This procedure is summarized in Algorithm 1. When *m* is large, this can result in considerable computational savings; although the trick is quite trivial to implement, it has not to our knowledge been employed for ERGM inference in prior work.

**Algorithm 1** Maximum Likelihood Inference for a Pooled ERGM Using Mean Values

**Input**: Observed data ***y***^*obs*^

1: Compute $\bar{g}\left(y^{obs}\right)=\frac{1}{m}\sum_{i=1}^{m}g\left(y_{i}\right)$

2: Find $\hat{\theta }=\text{arg}{\text{max}}_{\theta }\eta {\left(\theta \right)}^{⊤}\bar{g}\left(y^{obs}\right)-\psi \left(\eta \left(\theta \right)\right)$

3: Find $\hat{\text{Var}}\hat{\theta_{m}}=\frac{1}{m}{\left[\nabla \eta {\left(\hat{\theta }\right)}^{⊤}I\left(\hat{\theta }\right)\nabla \eta \left(\hat{\theta }\right)\right]}^{-1}$

**Output**: $\hat{\theta_{m}},\hat{\text{Var}}\hat{\theta_{m}}$

### 2.2 Conjugate maximum-a posteriori inference for ERGMs

We now consider the problem of IID pooled ERGM inference in the Bayesian setting. Given prior *π*(*θ*), we are interested in the posterior distribution of *θ*, *π*(*θ*|***y***^*obs*^),

$$\begin{array}{cc} \pi (\theta |y^{obs}) & =\frac{P_{\theta }\left(Y^{1}=y^{i},Y^{2}=y^{2},\cdots ,Y^{m}=y^{m}\right)\pi \left(\theta \right)}{\int P_{\theta }\left(Y^{1}=y^{1},Y^{2}=y^{2},\cdots ,Y^{m}=y^{m}\right)\pi \left(\theta \right)d\theta }\\ & \propto \pi \left(\theta \right)\prod_{i=1}^{m}P_{\theta }\left(Y^{i}=y^{i}\right). \end{array}$$ (7)

Our focus here is on conjugate priors, in the canonical exponential family context for which *η*(*θ*) = *θ*. In addition to their mathematical convenience, conjugate priors are attractive in the context of exponential families due to their interpretability (being able to be expressed in terms of prior “pseudo-data,” consisting of a prior “mean” and effective “sample size” expressed in the same units as the observed data), the fact that they admit natural non-informative limits, and their status as maximum entropy distributions [69]. To our knowledge, conjugate priors for ERGMs were first examined in the unpublished work of [70], who considered them along with a number of other ERGM prior specifications, but to date they have not been extensively studied. As we show, ERGM conjugate priors allow for extremely computationally efficient inference via their mean value representation. Moreover, there are particularly natural choices of weakly informative conjugate priors that are well-suited to regularization; we consider these in section 2.2.1.

For a canonical ERGM family with *η*(*θ*) = *θ*, conjugate priors take the following form [54]:

$$\begin{array}{c} \pi \left(\theta |\bar{\tau },n_{0}\right)=H\left(\bar{\tau },n_{0}\right)\;\text{exp}\;\{n_{0}{\bar{\tau }}^{⊺}\cdot \theta -n_{0}\psi \left(\theta \right)\}. \end{array}$$ (8)

Here, $\bar{\tau }$ are prior expected values of the vector of sufficient statistics, and *n*_0_ is a positive number that measures the confidence in those prior expectations, which can be viewed as the number of *pseudo-observations*’ worth of information (in units of observed graphs) contained in the prior; $H(\bar{\tau },n_{0})$ denotes the normalizing factor that makes $\pi (\theta |\bar{\tau },n_{0})$ a legitimate probability density function of *θ*. The existence of such distribution is ensured by [71], who showed (8) is normalizable provided that *n*_0_ > 0 and $\bar{\tau }$ lies in the interior of convex hull of the support of the measure *θ*. Substitute (8) for *π*(*θ*) in (7), we have

$$\begin{array}{cc} \pi (\theta |y^{obs},\bar{\tau },n_{0}) & \propto \text{exp}\{{\left[n_{0}\bar{\tau }+\sum_{i=1}^{m}g\left(y^{i}\right)\right]}^{⊺}\theta -\left(n_{0}+m\right)\psi \left(\theta \right)\}\\ & =\text{exp}{\left\{{\left[\frac{n_{0}\bar{\tau }+\sum_{i=1}^{m}g\left(y^{i}\right)}{n_{0}+m}\right]}^{⊺}\theta -\psi \left(\theta \right)\right\}}^{n_{0}+m}\\ & =\text{exp}{\left\{{\left[\delta \bar{\tau }+\left(1-\delta \right)\bar{g}\left(y^{obs}\right)\right]}^{⊺}\theta -\psi \left(\theta \right)\right\}}^{n_{0}+m}, \end{array}$$ (9)

where $\delta \equiv \frac{n_{0}}{n_{0}+m}$, taking values in [0, 1]. With (9), we note that an analytical form for the prior $\pi (\theta |\bar{\tau },n_{0})$ is not necessary for Bayesian inference, because the prior can be fully characterized by *δ* (or *n*_0_) and $\bar{\tau }$. Standard Bayesian theory tells us that the posterior expectation of ∇*ψ*(*θ*) is the Bayes estimate of *θ* with respect to quadratic loss [72], and is a weighted average of $\bar{g}\left(y^{obs}\right)$ and $\bar{\tau }$, with *δ* controlling the relative weight of contribution of the prior information. For any given prior hyperparameters $\left(\bar{\tau },n_{0}\right)$, as the sample size *m* becomes large, *δ*, the relative prior weight, approaches zero, and hence the sample-based information dominates the posterior.

Given a prior specified by *δ* and $\bar{\tau }$, the maximum *a posteriori* probability (MAP) estimate ${\hat{\theta }}_{m,\delta }^{MAP}$, is also Bayes estimate under a different choice of loss function (the 0–1 loss; see for example [72]). Note that since MAP is indeed the maximizer of the kernel of posterior density (9), we can employ the same arguments as in the derivation of (4), to obtain

$$\begin{array}{cc} {\hat{\theta }}_{m,\delta }^{MAP} & =\underset{\theta }{\text{arg}\;\text{max}}\;\text{exp}{\left\{{\left[\delta \bar{\tau }+\left(1-\delta \right)\bar{g}\left(y^{obs}\right)\right]}^{⊺}\theta -\psi \left(\theta \right)\right\}}^{n_{0}+m}\\ & =\underset{\theta }{\text{arg}\;\text{max}}\;\text{exp}\{{\left[\delta \bar{\tau }+\left(1-\delta \right)\bar{g}\left(y^{obs}\right)\right]}^{⊺}\theta -\psi \left(\theta \right)\}\\ & =\underset{\theta }{\text{arg max}}\;{\left[\delta \bar{\tau }+\left(1-\delta \right)\bar{g}\left(y^{obs}\right)\right]}^{⊺}\theta -\psi \left(\theta \right). \end{array}$$ (10)

It follows, then, that the pooled ERGM MAP estimator ${\hat{\theta }}_{m,\delta }^{MAP}$ is equal to the MLE $\hat{\theta }$ that would be obtained for a single pseudo-observation with sufficient statistics $\delta \bar{\tau }+\left(1-\delta \right)\bar{g}\left(y^{obs}\right)$.

Under standard regularity conditions, the posterior distribution $\pi (\theta |y^{obs},\bar{\tau },n_{0})$ becomes asymptotically Gaussian as *m* → ∞, according to the classical Berstein-von Mises theorem [73]. Following the same basic “mean value” procedure used in Algorithm 1 for obtaining the pooled ERGM MLE ${\tilde{\theta }}_{m}$, we are able to compute the MAP estimate ${\tilde{\theta }}_{m,\delta }^{MAP}$ by fitting an ERGM to a single ‘pseudo’-graph whose node set is the same as the observed networks but whose network statistics are taken to be equal to $\delta \bar{\tau }+\left(1-\delta \right)\bar{g}\left(y^{obs}\right)$. In addition to the MAP estimate, we can also obtain an estimate of the observed Fisher information $\hat{I}\left({\tilde{\theta }}_{m,\delta }^{MAP}\right)$, which is approximately the negative Hessian of log-posterior generated by product of the prior and the likelihood of a single ‘pseudo’-graph. However, the Laplace approximation of posterior distribution requires the Hessian of true log-posterior, which should be generated as by the product of prior and the likelihood of all actual observations. Note that the negative Hessian matrix *Q*_*m*,*δ*_(*θ*) of true log-posterior (9) can be approximated by $Q_{m,\delta }\left({\tilde{\theta }}_{m,\delta }^{MAP}\right)\approx \left(m+n_{0}\right)\hat{I}\left({\tilde{\theta }}_{m,\delta }^{MAP}\right)$ = $\frac{m}{1-\delta }\hat{I}\left({\tilde{\theta }}_{m,\delta }^{MAP}\right)$. *Laplace’s approximation* of the posterior yields [74] the following result,

$$\begin{array}{c} \theta |y^{obs}\overset{\cdot }{∼}N(\;{\tilde{\theta }}_{m,\delta }^{MAP},\;Q_{{}_{m,\delta }}^{-1}\left({\tilde{\theta }}_{m,\delta }^{MAP}\right)). \end{array}$$ (11)

We complete the approximation by noting that $\hat{I}\left({\tilde{\theta }}_{m,\delta }^{MAP}\right)={\text{Var}}_{{\tilde{\theta }}_{m,\delta }^{MAP}}g\left(Y\right)$, which can be obtained by Markov Chain Monte Carlo simulation [30].

Putting the pieces together, Algorithm 2 provides a simple procedure for performing MAP inference for pooled ERGMs under conjugate priors. We begin by specifying the prior parameters $\bar{\tau }$ and *n*_0_, and computing the mean data vector $\bar{g}\left(y^{obs}\right)$ and relative prior weight *δ*. The key steps are lines 3–4, which obtain the MAP estimate and associated approximate posterior variance-covariance matrix by performing the same calculations as are required for obtaining a single-graph MLE and its sample variance-covariance matrix: we simply fit to the posterior expectation $\delta \bar{\tau }+\left(1-\delta \right)\bar{g}\left(y^{obs}\right)$ instead of to an observed data value, and then adjust the information matrix to reflect the total posterior weight (prior pseudo-observations plus *m*). Not only does this allow us to perform inference for large-*m* data sets at no additional cost (as we did for the MLE), but it also allows us to perform Bayesian inference using algorithms and/or software implementations that were designed for maximum likelihood inference (or for first-order method-of-moments, which corresponds to maximum likelihood in this case) without additional modification.

**Algorithm 2** MAP Inference for a Pooled ERGM Using Mean Values

**Input**: Observed data ***y***^*obs*^, prior data expectation $\bar{\tau }$ and sample size *n*_0_

1: Compute $\bar{g}\left(y^{obs}\right)=\frac{1}{m}\sum_{i=1}^{m}g\left(y_{i}\right)$

2: Let *δ* = *n*_0_/(*n*_0_ + *m*)

3: Find ${\tilde{\theta }}_{m,\delta }^{MAP}=\text{arg}\;{\text{max}}_{\theta }{\left[\delta \bar{\tau }+\left(1-\delta \right)\bar{g}\left(y^{obs}\right)\right]}^{⊤}\theta -\psi \left(\eta \left(\theta \right)\right)$

4: Find $\hat{\text{Var}}{\tilde{\theta }}_{m,\delta }^{MAP}=\frac{1}{m+n_{0}}{\left[I({\tilde{\theta }}_{m,\delta }^{MAP})\right]}^{-1}$

**Output**: ${\tilde{\theta }}_{m,\delta }^{MAP},\hat{\text{Var}}{\tilde{\theta }}_{m,\delta }^{MAP}$

In addition to its computational convenience, we note that the posterior expected statistic $\delta \bar{\tau }+\left(1-\delta \right)\bar{g}\left(y^{obs}\right)$ has an intuitive geometric interpretation as a convex combination of the prior information and the observed information, with the respective weight being determined by the relative size of the prior weight *n*_0_ versus *m*. In particular, note that as *δ* → 0, we approach the MLE, while the prior becomes unchanged by the observed data in the limit as *δ* → 1. We examine this behavior in greater detail below. We also observe that so long as $\bar{\tau }$ lies in the relative interior of the convex hull of $\left\{g\left(y\right):y\in Y_{n}\right\}$, then ${\tilde{\theta }}_{m,\delta }^{MAP}$ exists (and is unique). This suggests the use of conjugate MAP to address a common practical problem in ERGM inference, namely the non-existence of the MLE when the observed statistics *g*(*y*^*obs*^) lie on the face of the convex hull of possible statistics. In such cases, there is a direction of recession within the parameter space, with respect to which the MLE diverges; often, however, such divergent parameter values arise from very minimal information, as when a small subset of vertices in a sparse graph have no ties to each other (leading to a divergence in the corresponding homophily term). Use of MAP inference with a small *δ* can improve performance in these cases by acting as a *regularizer*, shrinking in extreme parameter estimates that have little support from the likelihood without otherwise greatly altering the solution. We examine this further in Section 2.2.1.

#### 2.2.1 Conjugate prior specification

Conventional research on Bayesian analysis of ERGMs focuses on priors assigned on the natural parameter space (see e.g., [28, 29, 33]), whereas the ERGM conjugate prior here is actually specified in the mean-value parameter space. This has the potential advantage that prior parameters are specified in terms of hypothetical observables (i.e., graph statistics), which are both concrete and generalizable from previously observed data; for instance, it may be easier for the analyst to specify an expected mean degree for a hypothetical network belonging to a well-studied class (e.g., friendship nominations within high schools) than to specify prior mean parameter values *per se*. By turns, given an intuition regarding plausible parameter values, it is straightforward to obtain corresponding values of $\bar{\tau }$ by simulation. Here, we discuss some basic strategies for selecting reasonable prior parameters in practice, with the impact of prior choices being examined further in Section 3.2.

As discussed in Section 2.2, the specification of an ERGM conjugate prior consists of two components: the *a priori* expected sufficient statistics, $\bar{\tau }$, and the corresponding prior weight, *n*_0_. As with other exponential families, we may imagine this prior as arising from a situation in which we initially have no information regarding *θ* (in the sense of a limiting “flat” prior with *n*_0_ → 0), and then observe *n*_0_ IID graph draws with mean statistics $\bar{\tau }$; our resulting state of knowledge is then summarized by the corresponding conjugate prior. This “prior pseudo-data” interpretation makes the conjugate prior particularly easy to understand and communicate, and it can greatly facilitate sanity checking: for instance, if we observe that a proposed $\bar{\tau }$ value implies a mean degree far in excess of any value that could plausibly be observed in practice, then we are immediately aware of the need for refinement.

While prior specification is by nature problem specific, we here suggest several reasonable strategies for selection of $\bar{\tau }$. Where the analyst has access to a sample of networks, ***y***^*comp*^, that are similar to the network of interest (i.e., that are believed to have been produced by a similar generative process) setting $\bar{\tau }=\bar{g}\left(y^{comp}\right)$ is a natural informative choice; in this case, the posterior expectation of *g*(*Y*) is shrunk towards the prior population mean. In other cases, however, the analyst may lack such a sample, or may wish to posit a minimally informative prior that regularizes inference without strongly influencing the final estimate (a long-established tradition in Bayesian analysis, per e.g., [75–78], etc.). In this context, it is useful to consider the homogeneous Bernoulli graphs (in which each edge is an IID Bernoulli trial), as a basis for the prior distribution; proposed as early as [79], then later described independently by [80, 81] as the Gilbert-Erdős-Rényi model in graph theoretic research, the Bernoulli graphs also arise for typical (counting measure) ERGMs as the base case where all parameters other than that associated with the edge count are equal to 0. Given a prior expected degree $\bar{d}$ (chosen, for instance, on the basis of observations of similar networks, or from prior domain knowledge), we may then set $\bar{\tau }$ by (1) drawing a sample of IID Bernoulli graphs $Y_{p}^{Bern}$ with parameter $p=\bar{d}/\left(n-1\right)$, and then (2) setting the prior expectation $\bar{\tau }=\bar{g}\left(Y_{p}^{Bern}\right)$. (In some cases, it may also be feasible to derive the expected statistics analytically from *p*, in which case these values may be used directly; however, exact sampling of Bernoulli graphs is extremely efficient, and a Monte Carlo approach may be easier to implement in practice.) As the Bernoulli graphs coincide with the *de facto* null model against which estimated parameters are typically assessed, setting $\bar{\tau }$ to the Bernoulli graph expectation effectively shrinks estimates towards the null model (analogously to the use of a zero-centered Gaussian or other prior in the natural parameter space), making it a reasonable default choice when more refined information is not available.

We now turn to the prior weight (“pseudo-sample size”), *n*_0_. It is convenient to consider *n*_0_ via the relative prior weight, *δ* = *n*_0_/(*n*_0_ + *m*), which quantifies the contribution of the prior to the posterior mean statistics—the prior will dominate the data in determining the posterior when *δ* → 1 (i.e., *n*_0_ → ∞), whereas a more “objective” analysis which lets the data “speak for themselves” can be obtained by letting *δ* → 0 (i.e. *n*_0_ → 0, which as noted converges to the MLE). As noted above, a small-*δ* prior can also be viewed as a tool to regularize the model to avoid the extreme inferences resulting from data that is at or near the face of the convex hull of the sufficient statistics. While the impact of *δ* on the posterior mean of the sufficient statistics is self-evident from Eq 10, its effect in the natural parameter space is less obvious. We examine this numerically via simulation in Section 3.2.

## 3 Simulation studies

In this section, we conduct simulation studies to assess the behavior of the pooled MLE as *m* becomes large, and to examine how prior specifications affect conjugate MAP inference. To provide a realistic basis for evaluation, we base our simulated networks on Goodreau’s Faux Mesa High School (FMHS) data [64], a synthetic network based on proprietary data on attributions of friendship among students in a high shool in the southwestern United States [82]. The FMHS network represents simulated in-school friendships among the 205 students in the school, along with their individual attributes, and was constructed to preserve the structural properties of the underlying data set. For our study, we first fit an ERGM model to the FMSH network with the following three statistics as implemented in [67]: number of edges; uniform homophily by gender; and geometrically weighted edgewise shared partners (GWESP) (a common term for inducing triad closure), with the decay parameter λ fixed at 0.25. A detailed definition of above-mentioned network statistics is in S1 Appendix. Given the specified model, we first compute the MCMC MLE and treat the estimated coefficients as the networks’ “true” parameter values *θ*_0_ = (−5.885, 0.532, 1.867); we henceforth refer to this model (i.e., ERGM distribution) as M, from which we draw random samples for our simulation studies (i.e., Y∼M). All computations in this paper were carried out with the statistical environment R [83], using the statnet libraries for R [64, 84–86]. The ergm R library version 4.1.2 was used for all ERGM-specific computation, using default simulation and estimation settings except as otherwise noted.

### 3.1 Behavior of the MLE in pooled-likelihood inference

For our first study, we vary the sample size *m* and examine the observed coverage rates of nominal 95% confidence intervals for model parameters as a function of sample size. Specifically, for each value of *m*, we generate *K* = 1000 datasets of size *m* from M, performing pooled likelihood-based inference for each sample as discussed in Section 2.1. Respective burn-in and thinning intervals of 1 × 10^6^ and 2 × 10^5^ were employed for each simulated sample (for both data simulation and MCMC-MLE inference), with MCMC-MLE termination based on the ergm Hotelling criterion (an autocorrelation-adjusted *T*^2^ test of expected versus target statistics obtaining *p* > 0.5). Table 1 presents the observed coverage rates of nominal 95% confidence intervals based on the asymptotic distribution of the MLE, as estimated from the size-corrected Fisher information obtained from a pooled (single-graph) estimate.

**Table 1:** Pooled MLE bias, standard error, and coverage rates of 95% Wald confidence intervals as a function of sample size.

	edges			nodematch(Gender)			GWESP(0.25)		
*m*	Bias	(SE)	CP*	Bias	(SE)	CP*	Bias	(SE)	CP*
1	-0.0014	(0.138)	0.955	0.0035	(0.131)	0.950	-0.0097	(0.11)	0.949
2	-0.0049	(0.095)	0.946	-0.0022	(0.087)	0.950	0.0010	(0.078)	0.953
5	-0.0001	(0.06)	0.955	0.0019	(0.058)	0.937	-0.0022	(0.048)	0.947
10	0.0020	(0.043)	0.955	-0.0012	(0.04)	0.951	-0.0021	(0.035)	0.949
20	0.0006	(0.031)	0.946	-0.0004	(0.028)	0.951	-0.0007	(0.025)	0.935
30	0.0012	(0.027)	0.930	-0.0002	(0.023)	0.953	-0.0012	(0.022)	0.930
40	0.0005	(0.022)	0.942	-0.0010	(0.021)	0.939	0.0000	(0.018)	0.946
50	-0.0008	(0.02)	0.936	0.0000	(0.019)	0.932	0.0008	(0.016)	0.948
75	-0.0002	(0.017)	0.937	-0.0004	(0.015)	0.943	0.0005	(0.014)	0.943
100	0.0005	(0.015)	0.920	0.0004	(0.014)	0.933	-0.0006	(0.012)	0.940

* CP: Coverage Probability approximated by coverage rates of the simulated data

Table 1 shows the observed bias of the pooled MLE, as well as its standard error and the observed coverage rates of its nominal 95% CIs for all three model parameters under different sample sizes ranging from 1 to 100 graphs. Bias is negligible even for a single graph, declining to the levels of numerical noise once *m* is greater than 5–10. Likewise, efficiency (as measured by the standard error of the estimator) is high, and scales with $\sqrt{m}$ in the manner expected from asymptotic theory. It is also evident that CIs based on the asymptotic distribution of Eq 5 perform well, maintaining approximately nominal coverage rates over a wide range of sample sizes. As a practical observation, we note that the construction of such CIs is based on statistical uncertainty, and does not take into account numerical sources of error (arising, e.g., from imperfect optimization, Monte Carlo error, etc.). (This is a slight oversimplification, as the single-graph information matrix estimates here (produced by the ergm library) do incorporate some MCMC error correction. However, it is difficult to account for all sources of numerical error, and in any event the theory of Eq 5 does not address it.) As *m* → ∞, the statistical error becomes arbitrarily small, thus increasing the proportion of *de facto* error arising from numerical approximation; put another way, it is possible to enter a regime in which our inferential precision is limited by our ability to compute the MLE (and $\hat{I}$) rather than by the limits of our data. To ensure accurate coverage in such extreme-*m* scenarios, it may be necessary to adopt more stringent MCMC burn-in and thinning settings than are typically necessary for single-graph inference (where statistical uncertainty dominates), or to devise improved error estimates that better account for approximation error. That said, we do find excellent performance for sample sizes considered here, suggesting that the problem may be limited in practice. Further, we observe that the excellent coverage obtained for small *m* (even *m* = 1) provides practical validation of the traditional practice of using asymptotic confidence intervals in the *m* = 1 case; for a review of different types of ERGM asymptotics (and their relationship to classical results) see e.g., [35].

### 3.2 Prior weights and MAP inference

As discussed in Section 2.2.1, choosing the relative prior weight (*δ*) is an important aspect of the prior specification; while the choice of $\bar{\tau }$ can often be made based on either prior data or domain knowledge, the impact of *n*_0_ (hence *δ*) is less obvious. Here, we examine the impact of *δ* on the MAP estimate with a particular interest in identifying prior parameter values that are likely to serve as reasonable starting points for use in regularization. Our analysis looks first at the impact of *δ* on the MAP estimate itself (i.e., the extent of interpolation between the implicit prior natural parameter and the MLE), and then considers the effect of *δ* on the frequentist properties of the MAP estimate (bias, and the frequentist coverage of the posterior credible intervals).

To specify a prior, we first simulate homogeneous Bernoulli random graphs on the node set of the FMHS network, given expected mean degree fixed at the average degree of all the nodes in three comparable networks (i.e., Goodreau’s Faux Magnolia High School data, Faux Dixon High School data, and Faux Desert High School data [64]). The observed average degree across these data sets is 1.974, leading to an edge coefficient of $\text{log}\left(\frac{p}{1-p}\right)=\text{log}\left(\frac{\bar{d}}{n-\bar{d}-1}\right)=\text{log}\left(\frac{1.974}{205-1.974-1}\right)\approx -4.63$; for the Bernoulli family, the parameters for the other two terms are set to 0. (We note in passing that calibration of this kind should generally be done using mean degree rather than density, as mean degree is often close to size-invariant for comparable relations while density is not; see e.g., [26, 87].) We then calculate the average network statistics of 500 draws from this distribution, giving us the prior expected statistics $\bar{\tau }=\left(201.64,99.89,3.62\right)$. Since our focus is on *δ*, we fix our sample size at *m* = 1 and vary *n*_0_ to obtain the posterior inference under different values of relative prior weights. We perform MAP estimation on 1000 independent realizations of Y∼M for each choice of *δ*, comparing the resulting parameter estimates to their true values (*θ*_0_) to assess the bias of MAP estimate and the frequentist coverage probability of the 95% posterior credible intervals arising from the Laplace approximation to the posterior distribution.

**Fig 1:**
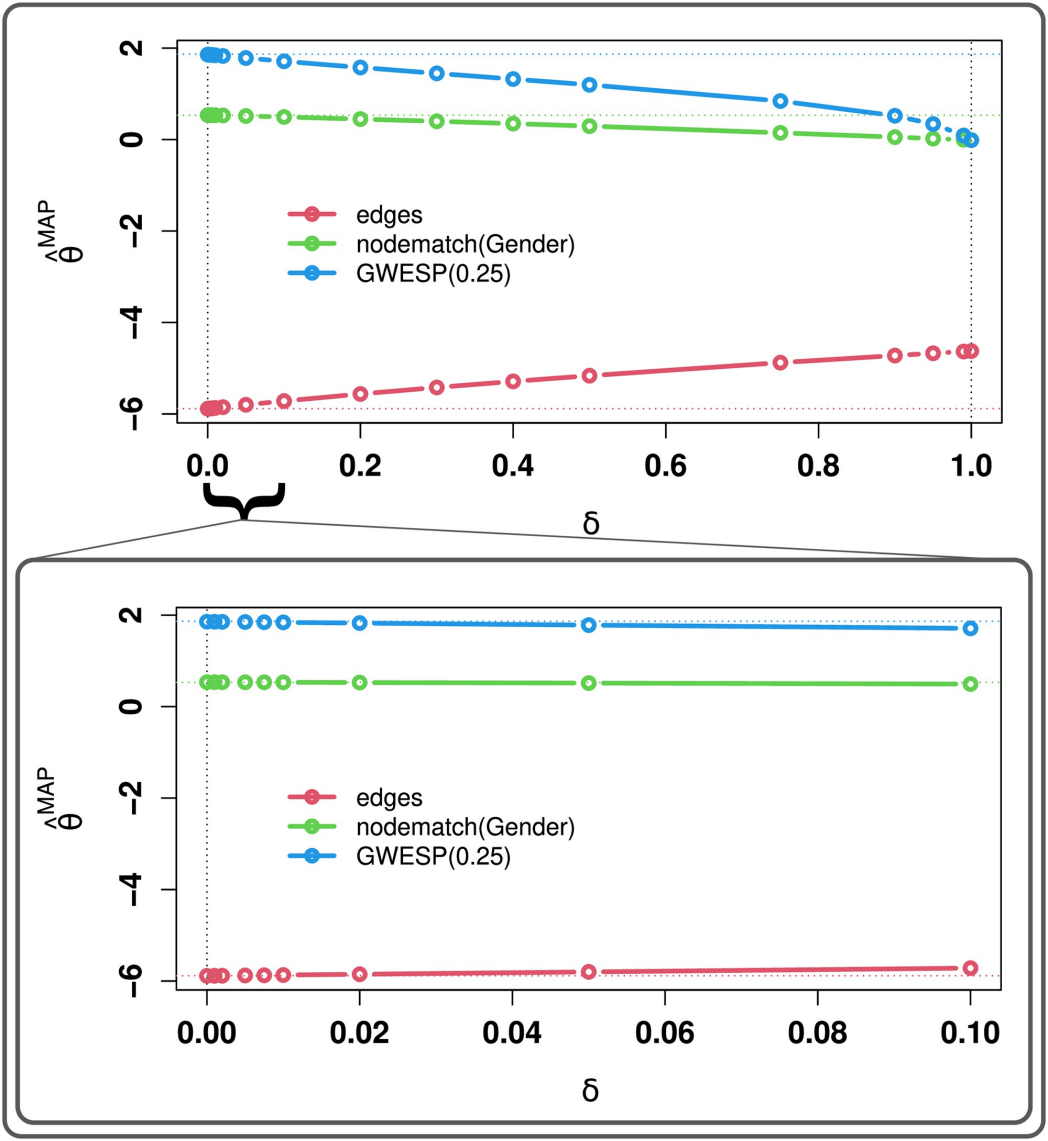
Average MAP estimates for Y∼M, by *δ*. Solutions interpolate between the natural parameters corresponding to the prior (*δ* = 1) and the MLE (*δ* = 0); shrinkage is nearly linear in *δ* near the non-informative limit (bottom panel). Dotted horizontal lines show data generating parameters.

We begin by examining the impact of *δ* on the MAP estimate. As noted above, the MAP estimate must interpolate between the natural parameter equivalent of $\bar{\tau }$ at *δ* = 1 and the MLE at *δ* = 0; equivalently, we may think of the conjugate prior as shrinking the estimate towards the (natural parameter equivalent of the) prior expectation. The detailed pattern of shrinkage is depicted in Fig 1, which shows that parameters change roughly linearly over most of the unit interval, with the most extreme changes occurring near *δ* = 1 (top panel). Importantly, shrinkage is approximately linear near the non-informative limit (*δ* → 0, bottom panel), suggesting that small differences in choice of *δ* do not have a large impact on the posterior mode (a convenient property when selecting minimally informative priors for regularization purposes). A more quantitative picture emerges from Table 2, which shows the mean MAP estimates for each parameter as a function of *δ*. We observe that choosing *δ* ≤ 0.02 yields estimates that are extremely close to the MLE (agreeing to 2–3 decimal places), while still placing sufficient weight on the prior to be useful for regularization (i.e., to ensure that the mean value parameter lies in the relative interior of the convex hull of possible statistics).

**Table 2:** Mean MAP estimates of model parameters under different relative prior weights when *m* = 1. (Data generating parameters *θ*_0_ = (−5.885, 0.532, 1.867); all standard errors for table entries less than 0.0044.).

*δ*	edges	nodematch(Gender)	GWESP(0.25)
0.0000	-5.8866	0.5352	1.8575
0.0010	-5.8854	0.5352	1.8560
0.0020	-5.8836	0.5349	1.8546
0.0050	-5.8784	0.5339	1.8501
0.0075	-5.8736	0.5325	1.8460
0.0100	-5.8692	0.5316	1.8423
0.0200	-5.8519	0.5273	1.8275
0.0500	-5.8003	0.5151	1.7830
0.1000	-5.7181	0.4936	1.7116
0.2000	-5.5636	0.4478	1.5763
0.3000	-5.4213	0.3994	1.4475
0.4000	-5.2891	0.3485	1.3225
0.5000	-5.1640	0.2941	1.1969
0.7500	-4.8787	0.1471	0.8424
0.9000	-4.7232	0.0531	0.5206
0.9500	-4.6739	0.0211	0.3394
0.9900	-4.6352	-0.0044	0.0895
1.0000	-4.6258	-0.0108	-0.0139

We now turn to the frequentist properties of the MAP estimate, as a function of *δ*. Here we compare the MAP estimate (and the 95% posterior intervals arising from the Laplace approximation) to the coefficients of true model M, which is *θ*_0_ = (−5.885, 0.532, 1.867); at the other extreme, we have the natural parameter equivalent of the location of the conjugate prior (−4.63, 0, 0). Table 3 shows the estimated bias and frequentist coverage probability for our simulation sample, as a function of *δ*. As can be seen, bias is minimal until *δ* ≈ 0.02, becoming substantial for *δ* > 0.1. Likewise, the 95% posterior intervals maintain good frequentist calibration until roughly *δ* ≈ 0.02, though coverage degrades rapidly thereafter. For regularizing/minimally informative applications, a choice of *n*_0_ ≈ 0.01 (giving the prior approximately 1% of the weight of a single graph observation) would seem to be a reasonable starting point.

**Table 3:** Frequentist properties of MAP estimates and the Laplace approximation for model parameters under different relative prior weights, with sample size fixed at *m* = 1.

		edges		nodematch(Gender)		GWESP(0.25)	
*δ*	$n_{0}^{†}$	Bias	(CP^‡^)	Bias	(CP^‡^)	Bias	(CP^‡^)
0.0000	0.000	-0.0014	0.955	0.0035	0.950	-0.0097	0.949
0.0010	0.001	-0.0003	0.953	0.0035	0.949	-0.0111	0.948
0.0020	0.002	0.0015	0.953	0.0032	0.952	-0.0126	0.949
0.0050	0.005	0.0067	0.953	0.0022	0.952	-0.0171	0.947
0.0075	0.008	0.0115	0.956	0.0008	0.952	-0.0212	0.942
0.0100	0.010	0.0159	0.956	-0.0001	0.949	-0.0249	0.945
0.0200	0.020	0.0333	0.946	-0.0044	0.948	-0.0397	0.930
0.0500	0.053	0.0848	0.900	-0.0166	0.952	-0.0842	0.894
0.1000	0.111	0.1670	0.733	-0.0381	0.942	-0.1555	0.654
0.2000	0.250	0.3215	0.157	-0.0839	0.911	-0.2909	0.098
0.3000	0.429	0.4638	0.001	-0.1323	0.818	-0.4196	0.000
0.4000	0.667	0.5961	0.000	-0.1832	0.604	-0.5447	0.000
0.5000	1.000	0.7211	0.000	-0.2376	0.269	-0.6703	0.000
0.7500	3.000	1.0064	0.000	-0.3846	0.000	-1.0248	0.000
0.9000	9.000	1.1619	0.000	-0.4786	0.000	-1.3466	0.000
0.9500	19.000	1.2112	0.000	-0.5106	0.000	-1.5278	0.000
0.9900	99.000	1.2500	0.000	-0.5361	0.000	-1.7776	0.000
1.0000	∞	1.2593	0.000	-0.5425	0.000	-1.8811	0.000

^†^ CP: Coverage Probability approximated by coverage rates of the simulated data; Laplace approximation is not applicable to *δ* = 1 because in that case the prior dictates the posterior inference, the posterior interval degenerates to a point mass

^‡^ *n*_0_ is the equivalent sample size contained in the prior given its relative weight *δ*

## 4 Applications

To demonstrate the pooled ERGM/conjugate prior analysis in practice, we provide two illustrative applications. The first is to the analysis of brain functional connectivity networks, where we seek a common model for brain structure across individuals. The second considers the use of ERGMs to model variation in protein structures obtained by X-ray crystallography, in this case using hen egg-white lysozyme (a widely studied reference protein). In each case, we show how the approach used here facilities the simultaneous analysis of multiple networks, and provides a fast and simple means of performing Bayesian inference.

### 4.1 Pooled ERGM analysis for brain functional connectivity networks

The study of group-based brain functional connectivity networks has become a topic of increasing interest in neuroscience, due the need to characterize both central tendencies and patterns of variation in interactions among brain regions. Importantly, it is of interest not only to measure specific or mean interactions, but to be able to characterize the distributions of interaction patterns arising under particular conditions, and/or within particular subpopulations. ERGMs have been identified as a promising tool for this purpose, due to their ability to assess how local brain network features give rise to the global structure, and due to their capacity to account for both heterogeneity and dependence among interactions [41, 88].

Brain functional connectivity networks often exhibit both functional segregation and integration [89], where functional segregation in the brain is the ability for specialized processing to occur within densely interconnected groups of brain regions, while functional integration corresponds to the ability to rapidly amalgamate specialized information from scattered brain regions. As an attempt to produce a model with appropriate network sufficient statistics that are able to capture those two concurrent opposing driving forces [42], proposed to first select the “best” metrics from a broader set of potential candidates identified in the literature using model selection techniques for ERGMs, then refit the networks of all subjects with those “best” metrics. They then employed the mean (respectively median) of the resulting individual estimates as estimates of a global, group-level “representative” whole-brain connectivity network model (which they refer to as a “mean” (respectively) “median” ERGM). This method of amalgamating models in the natural parameter space is straightforward and intuitive, but has several disadvantages: as shown in Eq 4, the appropriate pooling for a joint ERGM occurs in the mean value parameter space rather than the natural parameter space; separate estimation of an ERGM for each individual is computationally expensive (and, for the MLE, may encounter problems if some individuals’ networks have statistics that lie on the face of the convex hull of potential statistics); the sampling distributions of the amalgamated model estimates are unclear (especially in the median case); the amalgamated estimator is not in general consistent; and model selection by this approach does not exploit the joint likelihood (which may lead to an inferior pooled model).

By contrast, a pooled-ERGM approach provides a more principled and computationally efficient alternative to the mean/median ERGM approach. For large *n*, the properties of the pooled estimates and their confidence intervals are ensured by the large sample theory of exponential families, and as shown in Section 3.1 good results can be obtained with even modest numbers of graphs. Moreover, instead of having to fit each observed network separately, as proposed in [42] (with the risk that the MLE will not exist in one or more cases), exactly one ERGM fit is required (and the target statistics for that fit lie on the face of the convex hull only if all input networks do as well). Moreover, the ability to use conjugate-MAP inference for pooled ERGMs provides an inexpensive way of obtaining approximate Bayesian answers where desired, or (when viewing the prior as a regularizer) obtain regularized likelihood estimates. Here, we demonstrate all three approaches in the context of brain functional connectivity networks, building on prior work by [41, 42]. Due to the large number of model fits required for cross-validation, we use ergm’s stochastic approximation method for estimation in this section, with all Markov chains having a thinning interval of 5 × 10^4^ following 2 × 10^5^ burn-in iterations.

#### 4.1.1 Data

**Fig 2:**
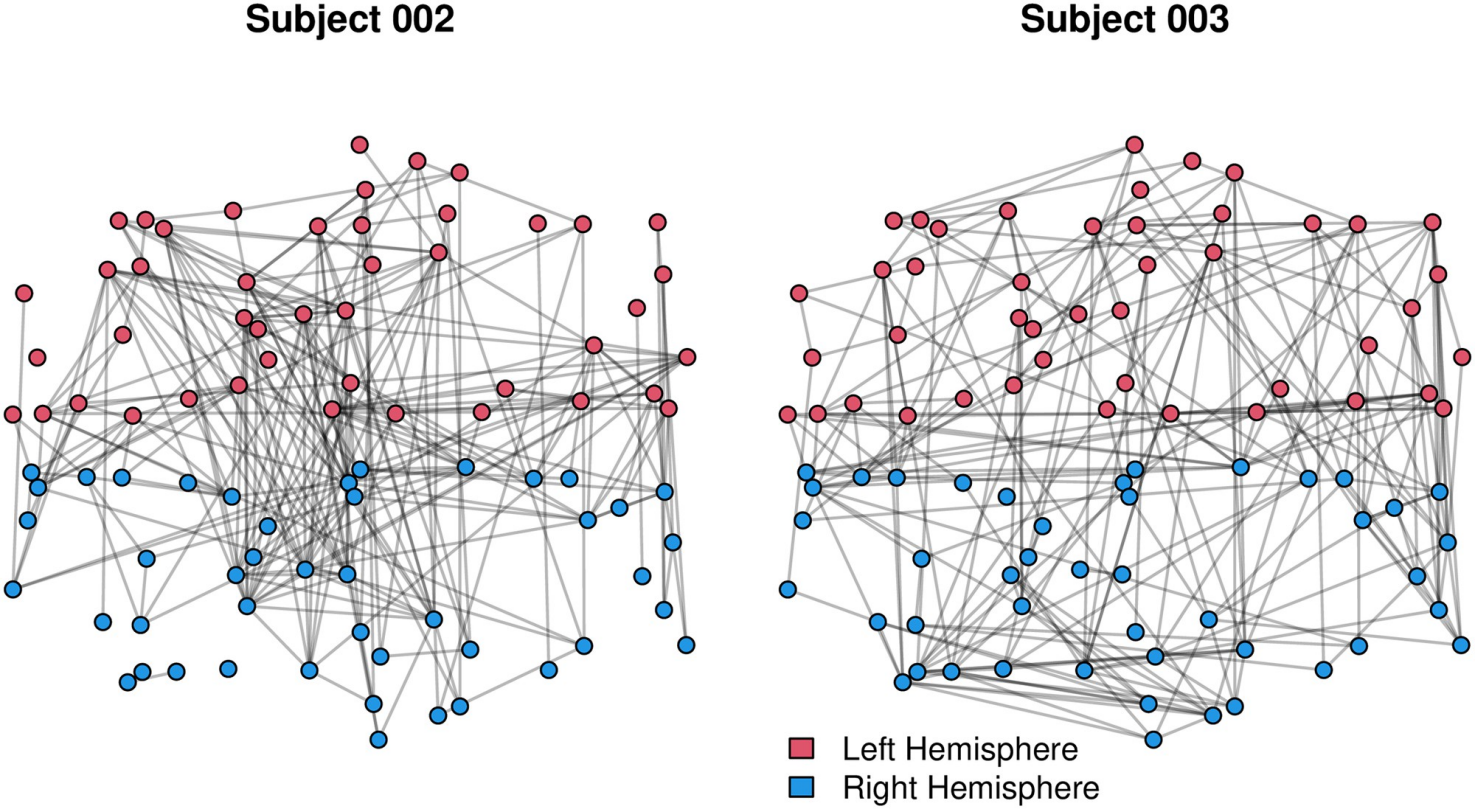
Brain connectivity networks of Subjects 002 and 003. Colors (red, blue) indicate the different hemispheres (left, right); node coordinates are based on an non-metric MDS [93] solution for distances between regions of interest.

We consider the data reported in [41, 42], which includes brain functional connectivity networks among 10 normal subjects (5 female, average age: 27.7 years old, standard deviation: 4.7 years) who were part (Subject No. 002, 003, 005, 008, 009, 010, 012, 013, 016, 021) of a larger functional MRI study of age-related changes in cross-modal deactivations [90]. Fig 2 depicts the brain connectivity networks of subjects 002 and 003, illustrating both common properties (e.g., clustering, increased probability of ties within brain regions) and heterogeneity across networks; here, we are interested in capturing this distribution via an ERGM form. Note that brain connectivity networks are defined on equivalent sets of nodes, which here correspond to 90 prespecified brain regions (ROIs—Regions of Interest), according to the Automated Anatomical Labeling Atlas (AAL) [91]. Each of these 10 brain connectivity networks is represented by binary adjacency matrix, in which element (*i*, *j*) denotes the presence or absence of a functional connection between node *i* and node *j*. The establishment of binary functional connections was done by thresholding the temporal correlation coefficient adjusted for motion and physiological noise (see [41, 92] for further details), and hence those brain networks are undirected by construction. The thresholds were selected by the original authors at the subject level to give each network a mean degree of $\frac{\text{log}(n)}{\text{log}(\bar{d})}\approx 2.8$, or equivalently $\bar{d}\approx n^{1/2.8}$, where *n* is the total number of nodes (here, *n* = 90).

Covariate information associated with these networks includes not only the nodal covariates *Hemisphere* and *Area*, but also an edge-level covariate for the spatial distance matrix among the ROIs (Mean: 76.28 mm, SD: 28.93 mm). The 90 regions are divided symmetrically across left and right hemispheres, with each hemisphere consisting of 28 areas as presented in Table 4.

**Table 4:** Number of ROIs in each area of the brain; names follow Simpson et al. (2011).

Area	Left Hemisphere	Right Hemisphere
Amygdala	1	1
Angular	1	1
Calcarine	1	1
Caudate	1	1
Cuneus	1	1
Fusiform	1	1
Heschl	1	1
Hippocampus	1	1
Insula	1	1
Lingual	1	1
Olfactory	1	1
Pallidum	1	1
Paracentral	1	1
ParaHippocampal	1	1
Postcentral	1	1
Precentral	1	1
Precuneus	1	1
Putamen	1	1
Rectus	1	1
Rolandic	1	1
Supp	1	1
SupraMarginal	1	1
Thalamus	1	1
Parietal	2	2
Cingulum	3	3
Occipital	3	3
Temporal	5	5
Frontal	9	9

#### 4.1.2 Model specification

Connectedness, local clustering and global efficiency were introduced as the key components in previous work on brain connectivity network modeling [41, 42], with the latter two being proposed as proxies for functional segregation and functional integration respectively. As such, their joint effects are modeled explicitly as a combination of network statistics: edge count (Edges), GWESP, and *geometrically weighted null shared partners* (GWNSP). Such a model specification yields a homogeneous ERGM that is permutation invariant [16, 35], which leaves covariate information underutilized, and in turn makes the estimation difficult and unstable due to multimodality of the distribution [65]. Similar to the multicollinearity issue in regression, it can be problematic to include two closely correlated network statistics in an ERGM model, and the presence of GWESP and GWNSP in previous models is found to be associated with convergence issue in the present case. Here, we thus modify and extend the homogeneous model used in prior work by incorporating node-level heterogeneity and distance effects associated with the spatial structure of the brain, along with a less collinear combination of GWESP and graphletCount(1) terms to capture dependence. Specifically, we include as covariates: a homophily effect for hemisphere (hemisphere-nodematch), as introduced in [43]; a mixing effect for brain regions (Area-nodemix) as a measure of the strength of interaction between the brain regions belonging to different areas of the brain; and a dyadic covariate that controls for spatial proximity (log.spatial.dist-edgecov), implemented by an effect for the log of the distance between regions (a common choice for modeling geographical effects, e.g., [94]). In addition to providing substantive insight into the drivers of connectivity, we also observe that such covariate effects also improve model performance by separating clustering and bridging due to physical brain structure from emergent network properties arising from dependence effects. The latter are captured by two effects. First, a GWESP term with decay fixed at 0.5 (GWESP, *ϕ* = 0.5 chosen based on pilot models) is included to capture residual tendencies towards endogenous local clustering net of controls, and second, a graphletCount(1) term [95] helps capture open two-path structure (aka *local bridging*) like that previously examined using GWNSP in the models of [41, 42].

#### 4.1.3 Results

Table 5 presents maximum likelihood estimates of model coefficients and associated standard errors for the group-based brain connectivity network model under pooling, enabling us to infer the extent to which each of the proposed effects shapes the overall distribution of networks across test subjects. (Predictive fit plots in S1 Fig) We see a positive and statistically significant parameter estimate for the GWESP statistic, indicating high levels of triadic closure net of spatial and anatomical features; this is compatible with the theory of functional segregation proposed in prior work. Likewise, we see that bridging is significantly disfavored (i.e., a negative effect for graphlet 1), suggesting that open triads tend not to persist (net of other factors). In estimating mixing effects, we aggregate all areas other than *Frontal* and *Temporal* to a single level as “Others” due to the small sizes of these regions, providing a tripartite mixing structure; Frontal-Frontal ties act as the reference category. We see inhibition of ties between different regions, and null or positive tendencies towards formation of within-area ties, which provides additional evidence for functional segregation. We note that these effects persist net of the overall inhibition of ties between more distant regions, with tie probability declining (*ceteris paribus*) as approximately one over the inverse of the distance between nodes. An important exception is the case of cross-hemispheric interactions, which are actually favored (the negative nodematch indicating that within-hemispheric interactions are disfavored relative to those that cross hemispheres). This can be viewed as an indicator of functional integration, with the need for coordination across hemispheres working against the general tendency against long-range ties. Care is required in the quantitative interpretation of the positive Edges coefficient, given the existence of log.spatial.dist-edgecov. Specifically, note that mean of pairwise distances of the ROIs is 76.28 mm and hence at the mean log(76.28)≈4.334, we have $\text{Pr}\left(Y_{i,j}=1|Y_{i,j}^{c}=y_{i,j}^{c}\right)=\frac{\text{exp}(1.193-1.081\times 4.334)}{1+\text{exp}(1.193-1.081\times 4.334)}\approx 0.029$, conditional on the rest of the graph and all other effects held at zero, meaning that the baseline conditional probability of observing an edge (not involved in the creation of other network statistics included in the model) between pairs of regions at the average distance is still very low, as expected for sparse graphs.

**Table 5:** Model parameter estimates and standard errors for pooled-ERGM analysis of brain functional connectivity networks. (For nodemix effects, Frontal-Frontal is the reference category).

Term	Estimate	(s.e.)	
Edges	1.193	(0.180)	****
GWESP, *ϕ* = 0.5	1.622	(0.044)	****
graphletCount(1)	-0.182	(0.008)	****
log.spatial.dist-edgecov	-1.081	(0.032)	****
hemisphere-nodematch	-0.337	(0.043)	****
Frontal.Others-nodemix	-0.270	(0.055)	****
Others.Others-nodemix	-0.061	(0.057)	
Frontal.Temporal-nodemix	-0.277	(0.106)	**
Others.Temporal-nodemix	-0.351	(0.074)	****
Temporal.Temporal-nodemix	0.457	(0.101)	****

**** *p* < 0.0001,

*** *p* < 0.001,

** *p* < 0.01,

* *p* < 0.05

### 4.2 Approximate conjugate Bayesian analysis of brain functional connectivity networks

In this subsection, we demonstrate how one can conduct approximate conjugate Bayesian analysis as introduced in Section 2.2 for the dual purposes of approximating full Bayesian analysis and regularization. The construction of the prior is crucial regardless of the ultimate purpose. We adopt the simulation-based approach of Section 2.2.1 to specify the prior by noting that $\bar{d}\approx n^{1/2.8}$ by construction (i.e., choice of correlation threshold) for all brain functional connectivity networks in this dataset, and thus we set $\bar{\tau }$ is set to be equal to the mean of network sufficient statistics under a Bernoulli graph with $p=\frac{\bar{d}}{n-1}\approx \frac{n^{1/2.8}}{n-1}\approx 0.056$ (*n* = 90). The selection of relative weight *δ* is subject to vary depending upon the purpose, which is explored and discussed in detail with examples.

#### 4.2.1 MAP estimation for the pooled model

In the absence of strong *a priori* information regarding almost all aspects of the brain functional connectivity networks except for the mean degree, it is advisable to incorporate weak prior information; we do this by assigning a small value to the hyper-parameter *δ*, in this case setting *δ* = 0.02. Given a specified prior, we conduct Bayesian inference based on Algorithm 2, the resulting parameters being shown in Table 6. (Predictive fit plots in S2 Fig).

**Table 6:** MAP estimates and posterior standard deviations, conjugate Bayesian analysis of brain functional connectivity networks. (For nodemix effects, Frontal-Frontal is the reference category).

Term	Estimate	(s.d.)	95% credible interval
Edges	1.277	(0.196)	(0.893,1.662)
GWESP, *ϕ* = 0.5	1.584	(0.042)	(1.502,1.665)
graphletCount(1)	-0.186	(0.008)	(-0.202,-0.170)
log.spatial.dist-edgecov	-1.070	(0.036)	(-1.141,-0.999)
hemisphere-nodematch	-0.334	(0.048)	(-0.427,-0.241)
Frontal.Others-nodemix	-0.326	(0.078)	(-0.478,-0.174)
Others.Others-nodemix	-0.112	(0.073)	(-0.255,0.032)
Frontal.Temporal-nodemix	-0.287	(0.126)	(-0.533,-0.040)
Others.Temporal-nodemix	-0.390	(0.087)	(-0.560,-0.220)
Temporal.Temporal-nodemix	0.466	(0.120)	(0.231,0.700)

The parameter estimates from the Bayesian analysis are very similar to those of the pooled MLE, supporting the same qualitative conclusions. However, imposing a prior on the parameter vector permits interpretation of the results in terms of Bayesian answers, which may be useful in some settings; we may also use the Laplace approximation to sample from the approximate posterior, enabling us to obtain, for instance, posterior predictive distributions for network properties that take into account uncertainty in the model parameters.

#### 4.2.2 Regularizing ERGMs with MAP

As noted above, the MLE for the natural parameter of an exponential family distribution does not exist when the observed sufficient statistics lie on the relative boundary of *C*, the convex hull of the set of possible values of sufficient statistics. A common case of this type in ERGM modeling arises when mixing or differential nodematch parameters are specified for networks containing many small subgroups; if any of the associated statistics are equal to 0 (e.g., there are no observed ties between two groups), then the likelihood has no finite maximizer with respect to the respective directions in the natural parameter space. In the context of the brain connectivity networks, we observe that there are many small areas containing few nodes, potentially leading to such a circumstance. For instance, consider an extension of our previous model intended to quantify the mixing pattern between nodes in the *Occipital* and *Cingulum* areas; we may do so by augmenting $M_{1}$ with nodemix terms involving Occipital and Cingulum, with all other terms in the model unchanged. We denote this model as $M_{2}$. It happens, however, that there there are no edges observed between Occipital and Cingulum for any of the networks in the dataset, and hence the vector of mean observed network sufficient statistics is no longer located in *rint*(*C*) (as the Occipital.Cingulum-nodemix value of 0 is smallest possible value that can be obtained). From an optimization perspective, we are unable to obtain a finite estimate for model coefficients of this augmented model, because the likelihood can always be further optimized by letting the vector of candidate estimates of model coefficients move towards the direction of recession. Statistically, this reflects the non-existence of the MLE. We now show such issues can be resolved by incorporating an appropriate conjugate prior into the inference to regularize the model and thus avoid extreme inferences on model parameters.

We construct a conjugate prior in the form of (8), where hyper-parameter $\bar{\tau }$ is determined by calculating the mean of network sufficient statistics observed on 1000 independent random realizations of Bernoulli random graphs with *p* = 0.056. As our goal here is regularization, we view the prior as a convenient penalty function (rather than as a formal statement of prior knowledge), and treat *δ* as a hyperparameter subject to optimization. Given our pooled setting, it is natural to evaluate model performance by cross-validation (CV); specifically, we vary *δ* (or, equivalently, the prior sample size *n*_0_), computing the expected squared Hamming error for each graph under leave-one-out CV based on 1000 draws from each simulated model, and select the value that minimizes the expected loss on the held-out networks. The Hamming error (i.e., the expected number of edge differences between a predicted draw from the model and the observed network) is a straightforward and interpretable metric for models on labeled graphs, where specific connections (as opposed to, e.g., general network properties) are meaningful and where there is sufficient covariate information to make prediction possible; in the case of the brain functional networks, where the vertices have distinct anatomical and functional significance that is conserved across subjects, optimizing the ability of the model to make edgewise predictions is a reasonable goal. We note, however, that other choices are also possible, depending on one’s objectives: for instance, an obvious alternative is the squared error in the predicted sufficient statistics, a quantity more closely related to the MLE. An attractive feature of CV is thus the freedom of the analyst to tune the model based on the needs of the problem at hand.

The results of the hyperparameter tuning process are shown in Table 7. As expected, the unregularized MLE (*n*_0_ = *δ* = 0) yields suboptimal performance, with improvements obtained until *n*_0_ = 0.004 (*δ* = 0.0004). Further increases in prior weight (here interpreted as the strength of the penalty function) result in diminished performance, as the fitted model is drawn towards the prior mean. We thus select *δ* = 0.0004 for subsequent analysis.

**Table 7:** Leave-one-out cross validation error for regularized inference on $M_{2}$ as a function of *n*_0_.

*n*_0_	*δ*	CV Error
0.000	0.0000	612255.803
0.001	0.0001	616388.530
0.002	0.0002	611733.729
0.004	0.0004	610657.580
0.008	0.0009	617267.178
0.016	0.0017	616974.051
0.031	0.0035	620343.917
0.062	0.0069	610740.156
0.125	0.0137	612219.087
0.250	0.0270	614275.772
0.500	0.0526	618156.374
1.000	0.1000	625175.603
2.000	0.1818	636375.903
4.000	0.3077	650507.803
8.000	0.4706	670991.412

We may now perform penalized maximum likelihood inference, using the tuned conjugate prior as a regularizer. Table 8 shows the corresponding parameter estimates, standard errors, and significance levels for model $M_{2}$. (Predictive fit plots in S3 Fig) As expected, the results for the shared effects (triangulation, spatial interaction, bridging, and hemispheric interaction) after breaking out the additional brain areas remain very similar to what was seen in the unregularized MLE for the collapsed model, though we now have a more complete description of the mixing pattern among localized areas. Importantly, we also observe that the Occipital/Cingulum parameter (for which the MLE does not exist) is now well-characterized. As we would expect from the fact that none of the observed networks had Occipital/Cingulum ties, the estimated coefficient is significantly negative; however, the magnitude is now plausible (and in line with the other observed effects).

**Table 8:** Model parameter estimates and standard errors for regularized inference on $M_{2}$.

Term	Estimate	(s.e.)	
Edges	1.986	(0.224)	****
GWESP, *ϕ* = 0.5	1.623	(0.045)	****
graphletCount(1)	-0.166	(0.007)	****
log.spatial.dist-edgecov	-1.051	(0.036)	****
hemisphere-nodematch	-0.328	(0.044)	****
Cingulum.Frontal-nodemix	-1.109	(0.142)	****
Frontal.Frontal-nodemix	-1.006	(0.128)	****
Cingulum.Occipital-nodemix	-2.311	(0.351)	****
Frontal.Occipital-nodemix	-0.638	(0.189)	***
Occipital.Occipital-nodemix	-0.135	(0.153)	
Cingulum.Others-nodemix	-1.310	(0.130)	****
Frontal.Others-nodemix	-1.370	(0.129)	****
Occipital.Others-nodemix	-1.290	(0.136)	****
Others.Others-nodemix	-1.069	(0.124)	****
Cingulum.Temporal-nodemix	-1.325	(0.266)	****
Frontal.Temporal-nodemix	-1.278	(0.153)	****
Occipital.Temporal-nodemix	-1.767	(0.374)	****
Others.Temporal-nodemix	-1.340	(0.131)	****
Temporal.Temporal-nodemix	-0.514	(0.148)	***

****p < 0.0001,

***p < 0.001,

**p < 0.01,

*p < 0.05

Finally, we note in passing that, while this final model captures a number of aspects of brain network structure, more improvement seems possible. As seen in S3 Fig, the model is fairly accurate in recovering observed triad census and degree structure (nearly all observed values falling within the 95% simulation intervals), but somewhat underestimates both mean geodesic lengths and the breadth of the ESP distribution. Thus, the model might be satisfactory for investigation of local structure, but less so for larger-scale structure (including heterogeneity in triadic clustering throughout the brain). For applications of this latter sort, further elaboration or alternative parameterizations may be desired. We revisit the question of tradeoffs between model complexity and predictive performance in Section 5.3.

### 4.3 Analysis of lysozyme structure networks via pooled ERGMs

The functions of proteins and other macromolecules are heavily influenced by their three-dimensional structure. With the increasing sophistication of both experimental technique and molecular modeling, new methods for analyzing the growing body of protein structure data are of increasing interest. Network analytic methods have emerged as particularly useful tools for this purpose, providing a rich representation for topological complexity while still offering substantial coarsening relative to atomistic structure. Among other applications, network representations of protein structure have been used to identify functionally important residues [96], summarize protein dynamics [97], identify functionally significant sub-units [98], distinguish active site conformations [7], and characterize structural differences between protein families [99].

One potential application of ERGMs in the context of protein structure is the characterization of variation within structures of the same protein (either in equilibrium, or in different functional or measurement contexts). ERGMs were first applied to protein structure networks by [95], who used them to control for intrinsic molecular features (e.g., chain membership) while testing hypotheses regarding fold-specific structure. In more recent work, ERGMs have been employed to characterize transient structure in intrinsically disordered proteins [40], and to model protein aggregation [8, 100]. Here, we consider the problem of characterizing variation in measured protein structures obtained via X-ray crystallography (the primary workhorse technique of modern structural biology). While it is common to treat globular proteins as having a native fold associated with a single three-dimensional structure obtained via crystallographic methods (or, more rarely, Nuclear Magnetic Resonance, neutron scattering, or cryo-EM), proteins in solution are extremely dynamic; even in a crystallographic context, repeated crystallization of the same protein will often yield slightly different structures. (In fact, the same crystal frequently contains several distinct conformations within a single asymmetric unit.) Currently, this variation is not well-characterized, and is often ignored (with a single conformation selected as “the” structure of the protein). Statistically, it is natural to think of these observed structures as being drawn from a broader distribution of low-energy conformations [101, 102], and to attempt to model this distribution using the measured conformations.

**Fig 3:**
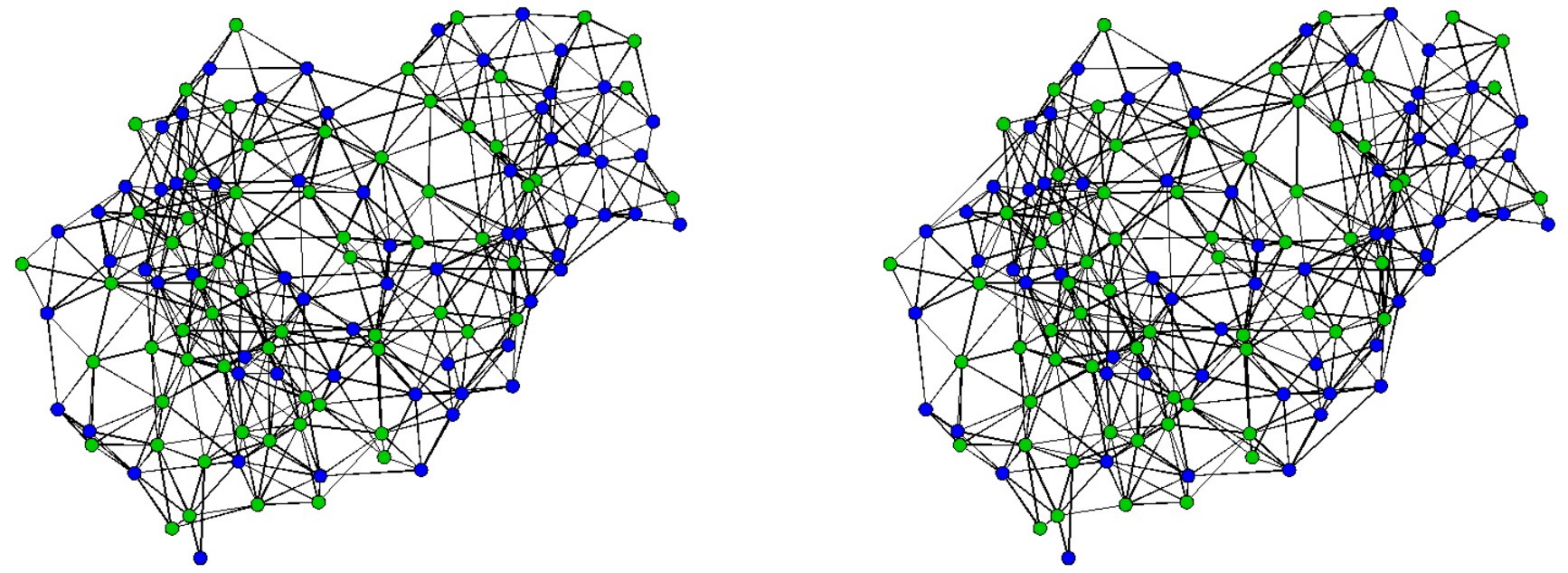
2D representation of Lysozyme structure networks (PDB 1AKI [104]; Left), (PDB 1BHZ [105]; Right). Colors distinguish nonpolar (green) versus polar (blue) residues; node coordinates determined via topology and are not based on physical position.

**Fig 4:**
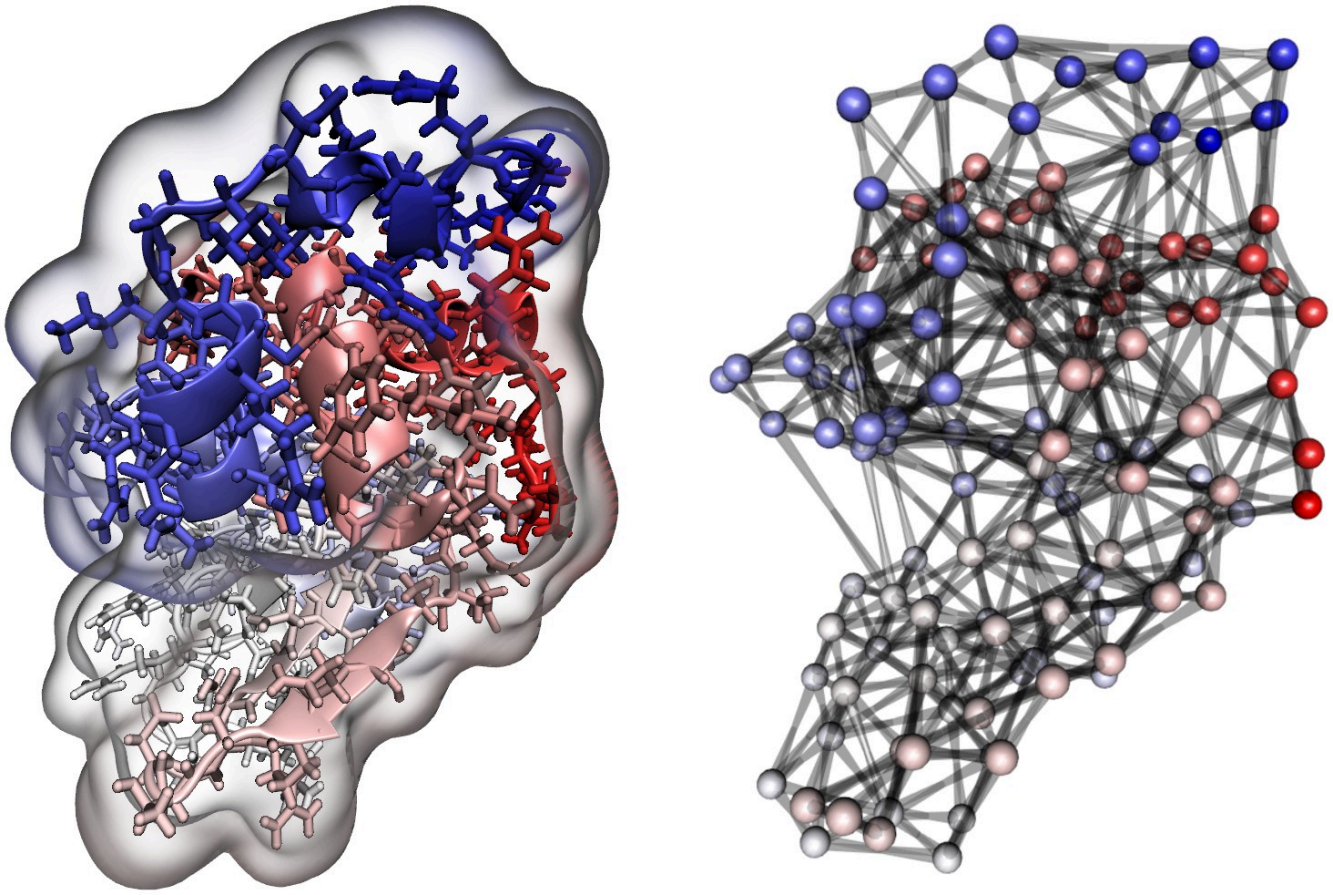
3D representations of lysozyme (PDB 1AKI [104]). (Left) Molecular representation, showing backbone (ribbon), side chains, and surface; residues colored by index. (Right) PSN representation in similar orientation, with vertices positioned by C*α* coordinates and colored by index.

Here, we apply this notion to observed variation in crystal structures of hen egg-white lysozyme (a widely used reference protein in biophysical research). Lysozyme (N-acetylmuramide glycanhydrolase), is an enzymatic antimicrobial agent produced as part of the innate immune system. A glycoside hydrolase, lysozyme attacks polysaccharides within bacterial cell walls, compromising their integrity and ultimately causing cell lysis; as such, it is produced in large quantities in settings where bacterial growth must be discouraged (e.g., eggs, tears, milk). Our data consist of network representations of 66 independently solved lysozyme structures, each of which is formed from 129 residues (i.e., amino acids) constituting the main chain of wild type hen egg-white lysozyme (residues 19–147 of Uniprot B8YK79). Atomistic protein structures were obtained from the Protein Data Bank (PDB; https://www.rcsb.org/pdb/home/home.do), with the search query limited to X-ray crystallography structures containing only the 129-residue main chain with no modified or substituted residues, missing residues, ligands, or other complexes. Where more than one distinct conformation appeared in the asymmetric unit, each was isolated and treated as a separate conformation for purposes of analysis. Each isolated protein structure was protonated using REDUCE [103], with the resulting coordinates employed to generate a residue-level protein structure network (i.e., an undirected adjacency matrix of size 129 × 129) according to the pairwise distances among residues—any pair of residues is considered to be adjacent if they contain respective atoms that are closer together than 1.2 times the sum of their respective van der Waals radii. Two representative lysozyme structure networks are displayed in Fig 3; while the conformations are very similar, they do show subtle differences (compare e.g., the residues in the top right). A 3D molecular structure of lysozyme is shown in Fig 4, together with the equivalent protein structure network (PSN).

#### 4.3.1 Model specification

**Model terms**: Our model specification includes three categories of effects: covariates relating to residue properties that enhance or inhibit interaction; “contextual” covariates relating to the overall fold of the protein; and dependencies among contacts arising from steric and other effects. Beginning with the first group, we add a Coulomb-like term for interactions based on nominal residue charge, ChargeMatch-edgecov, coded as 1 for pairs with complementary charges, -1 for pairs with non-complementary charges (i.e., positive/positive or negative/negative), and 0 otherwise. We include two terms for Polar/Polar (PolPol-edgecov) and Nonpolar/Nonpolar (NPolNPol-edgecov) residue pairs, respectively, accounting for the fact that the two affinities are non-identical. To account for the distinctive interaction patterns of aromatic residues, we include an overall effect for interaction by aromatics (Aromatic-nodecov) as well as an effect for pairwise interactions among aromatic residues *per se* (referred to mnemonically as PiStack-edgecov). Finally, we account for the greater contact potential of larger residues by incorporating a term for residue surface area (SurfaceArea-nodecov).

With respect to the second class of effects, we first observe that distance along the protein backbone is an important predictor of interaction, and we hence include the logged backbone distance as an edgewise covariate (logBBDist-edgecov); separately, we also incorporate adjacency along the backbone as a support constraint (reflecting the fact that each residue is covalently bound to its backbone neighbors). Because our objective is to model variation in folded lysozyme structures (and not predicting the fold *de novo*), we incorporate an effect for the average distances among residues over all structures. Specifically, we encode the log of the mean distance between alpha carbons (C*α*) for every residue pair (taken over all structures) as an edge covariate (logMeanDist-edgecov), expressing the intuition that residues that are on average spatially proximate in folded lysozyme are more likely to be adjacent in any particular structure. To account for the fact that surface residues have solvent and/or crystal contacts that are not captured by the structure (resulting in a lower mean degree within the PSN), we also include the mean C*α* distance from the coordinate center as a nodal covariate (meanCADist-nodecov). To adjust for differences in the ability of larger or bulkier residues to form contacts at longer C*α* distances, we also add respective product terms (i.e., interaction effects in a statistical rather than relational sense) between the aromatic and surface area statistics and the log C*α* distances (logMeanDistAro-edgecov and logMeanDistSurf-edgecov).

Finally, we consider terms relating to the interdependence among contacts. To model the fact that each of a residue’s existing contacts increases the difficulty of forming new contacts, we include a 2-star term (2-stars); likewise, we include a triangle term (Triangles) to account for the increasing difficulty of forming large cliques. (While both such terms are rarely used in social network settings due to their propensity to produce degenerate models when their associated coefficients are positive, these terms can be important for capturing geometric constraints in physical systems; since the associated coefficients are generally negative in these cases, they do not lead to runaway clique formation.) Although large cliques are strongly suppressed by packing constraints, PSNs *are* however highly triangulated. We thus combine the (hypothesized negative) triangle term with a GWESP term (here using a decay parameter of 0.8 identified by a pilot fit to a single graph). Finally, we note in passing that, while we do not do so here, it is possible to add maximum degree constraints that place limits on the maximum number of contacts per residue (to reflect steric constraints). Pilot analyses showed that, in this model, residues did not show unrealistically high contact rates, and imposing constraints did not affect the results. For computational simplicity, we thus do not employ them. However, this may be important in other systems, and should be considered for models that show unrealistically high contact rates.

**Prior specification**: To specify the prior for conjugate MAP, we begin with the approximation that the mean degree for a fully buried core residue will be approximately 12 (based on a standard sphere packing approximation; see e.g. [106]). In practice, however, many potential contacts are “lost” due to residues’ not being completely surrounded by other residues (i.e., on the surface). To approximate the fraction of possible contacts that are “lost” in this way, we begin by approximating the expected surface area of the protein that would be used for residue-residue contacts if all residues were buried; paradoxically, this is the surface area of the fully unfolded protein. [107] show that the empirical model

$$A_{u}\approx 1.48M+21$$

provides an excellent approximation to the unfolded surface area of monomeric proteins (where *A*_*u*_ is the surface area in squared Angstroms, and *M* is the molecular mass in Daltons). For the surface area of a folded protein, they likewise report the model

$$A_{f}\approx 6.3M^{0.73}$$

(with the same units as above). We may approximate the fraction of possible contacts “lost” to solvent in the folded protein as *A*_*f*_/*A*_*u*_, and thus approximate the expected degree by

$$\bar{d}\approx 12\left(1-\frac{A_{f}}{A_{u}}\right).$$

For lysozyme, we have *M* = 14.3kDa, giving us *A*_*f*_ ≈ 6803.554 Å^2^, *A*_*u*_ ≈ 21185 Å^2^, and $\bar{d}\approx 8.15$ (i.e., about 32% of potential residue contacts are predicted to be lost). Although obtained entirely via *a priori* considerations, we note that this expected degree is quite close to the observed degree for the lysozyme structures in our sample (8.32), suggesting that it is indeed a reasonable choice. To obtain $\bar{\tau }$, we simulate 1000 conditional Bernoulli graph draws with mean degree $\bar{d}$, subject to the constraint that all backbone-adjacent residues are tied, and take $\bar{\tau }$ equal to the means of the sufficient statistics for the sample.

To set the prior weight (*n*_0_, and hence *δ*), we observe that our prior information is fairly vague, and we would want the data to outweigh the prior even for a single graph observation. We thus set *n*_0_ = 0.1, making the prior weight equivalent to one tenth of a single graph observation. For our data set, with *m* = 66, this implies a net prior weight of *δ* ≈ 0.0015.

#### 4.3.2 Results

We perform conjugate MAP inference for the pooled ERGM model on the 66 lysozyme PSNs, using the above-specified model; estimation was performed using **ergm** under default settings incorporating the backbone-adjacency support constraint. The resulting parameter estimates are provided in Table 9. The model parameters can be interpreted based on the conditional log-odds of an edge between two nodes *i* and *j*, bearing in mind that many effects are necessarily simultaneous. For example, while the coefficient for the edges term is positive, it should be interpreted in the context of both mean spatial distances and sequence distances between residues. For example, the log mean distance between the C*α*s of residue 4 and residue 9 is 2.143, and their log backbone distance is log(5) = 1.6. Ignoring all other effects, then the conditional probability of *Y*_4,9_ = 1 based solely on these three terms would be [1 + exp[−(34.213 − 19.173(2.143) + 0.293(1.6))]]^−1^ ≈ 0.002, indicating a low conditional probability of observing an edge; in practice, of course, all terms contribute simultaneously.

**Table 9:** Conjugate Bayesian analysis of Lysozyme structure networks.

Term	Estimate	(s.d.)	95% credible interval
Edges	34.213	(0.0309)	(34.152, 34.274)
ChargeMatch-edgecov	0.229	(0.0654)	(0.101, 0.357)
NPolNPol-edgecov	0.760	(0.0296)	(0.702, 0.818)
PolPol-edgecov	0.186	(0.0319)	(0.124, 0.249)
Aromatic-nodecov	-1.091	(0.0122)	(-1.115, -1.067)
PiStack-edgecov	-0.752	(0.0849)	(-0.918, -0.585)
SurfaceArea-nodecov	-0.040	(0.0006)	(-0.041, -0.038)
logBBDist-edgecov	0.293	(0.0110)	(0.272, 0.315)
meanCADist-nodecov	-0.040	(0.0028)	(-0.045, -0.035)
logMeanDist-edgecov	-19.173	(0.0643)	(-19.299, -19.047)
logMeanDistSurf-edgecov	0.024	(0.0003)	(0.023, 0.024)
logMeanDistAro-edgecov	0.646	(0.0128)	(0.621, 0.671)
GWESP, *ϕ* = 0.8	1.444	(0.0385)	(1.368, 1.519)
2-stars	-0.046	(0.0058)	(-0.057, -0.035)
Triangles	-0.829	(0.0253)	(-0.879, -0.780)

As Table 9 shows, all three types of mechanisms play a role in predicting lysozyme network structure. Electrostatic and polar effects act as expected, with complementary charges increasing conditional tie probability and homophily for non-polar residues; although the posterior strongly favors homophily among polar residues, this effect is notably weaker than for the non-polar case.

Aromatic residues at first blush seem to have a lower baseline contact probability (with an additional negative effect for *π*-stacking), but these “intercept effects” must be weighed against the reduction in the C*α* distance penalty for these residues. Let *i* and *j* be residues *d* Å apart, such that *i* is aromatic and *j* is not. Then the total effect of the Aromatic, *π*-stacking, and Aromatic distance effect terms on the conditional log odds of an *i*, *j* edge is approximately −1.091 + 0.646 log *d*; this enhances tie probability for *d* > 5.4Å, only suppressing it at close range. Likewise, for aromatic-aromatic pairs, the corresponding total effect is approximately −1.091 − 0.752 + 2(0.646) log *d* = −1.843 + 1.292 log *d*, which becomes favorable for *d* > 4.2Å. We would expect mixing among aromatic residues to be favored on physical grounds, and indeed this is true for residue pairs beyond 3.2Å. Overall, we thus see that interactions with aromatic residues are generally favorable (with aromatic-aromatic mixing especially favorable) except at very close range, with these residues particularly likely to interact with other residues over longer distances. Very short-range interactions are somewhat hindered for these residues, however, plausibly due to steric effects.

A similar effect is seen for residue size, with surface area having a negative main effect combined with a greater propensity for longer-range interaction; for residues *i* and *j* with respective surface areas *s*_*i*_ Å^2^ and *s*_*j*_ Å^2^ at distance *d* Å, the total effect of the surface area terms on conditional log odds is −0.04(*s*_*i*_ + *s*_*j*_) + 0.024(*s*_*i*_ + *s*_*j*_) log *d*, which becomes positive for *d* > 5.3 Å. For reference, the mean non-covalent nearest neighbor distance is approximately 3.8 Å, and the second-nearest is approximately 5.1 Å, so bulk is a positive interaction predictor for the vast majority of potential interactions. The minor inhibitory effect at very small distances, like that of aromaticity, may reflect steric hindrance.

Similar subtlety is seen in the case of the mildly positive effect of backbone distance—net of spatial distance—likely reflecting the tendency of the backbone to fold back on itself (creating strong bridges between parts of the protein that are distinct in sequence space). Note that, marginally, we find that contact probabilities fall off as roughly *BBDist*^−5/4^, so this softening effect should not be confused with a net tendency for tie probability to increase with backbone distance. Rather, we find that when sequence-distant residues happen to be spatially proximate, they are particularly likely to be in contact. Less nuance is needed to interpret the effect of distance from the origin, or of the mean C*α* distance between residues: both inhibit contact. The latter effect is, as has been observed, very large, in keeping with the constraints of a folded protein. Finally, we observe that net of everything else, existing contacts have an inhibitory effect of new ones (the negative 2-star parameter), cliques are strongly suppressed (negative triangle parameter), and there is an overall tendency towards triangulation net of clique suppression (positive GWESP parameter). To understand how these latter two effects combine, it is useful to consider the net change in the conditional log odds of adding a first, second, third, etc. shared partner to an adjacent residue pair (holding all other effects constant). The (base or direct) GWESP effect for the *k*th ESP is here 1.44exp(0.8)(1 − (1 − exp(−0.8))^*k*^) = 3.20(1 − 0.55^*k*^)) for *k* > 0 (or 0 otherwise); such a configuration also involves *k* triangles (with a total weight of −0.83*k*). The net contribution of these terms to the log odds of going from *k* to *k* + 1 shared partners is then the difference in the effect sums; for adding the first shared partner, this yields a net contribution of 0.62 (strongly favorable), falling to -0.03 for the second (approximately neutral), -0.39 for the third (unfavorable) and -0.59 for the fourth (strongly unfavorable). These penalties continue to increase, approaching the limiting value of -0.83 (the triangle coefficient). (Note that adding a shared partner also adds at least one 2-star (possibly as many as three, depending on the assumed baseline conformation), reducing the log odds contribution by an additional -0.05 to -0.15. This is, however, a much smaller effect). Although not often used together, a triangle term can usefully combine with GWESP in cases like this where triangle formation must shift from favorable to unfavorable as more shared partners accumulate, a phenomenon that may also manifest in other systems.

**Fig 5:**
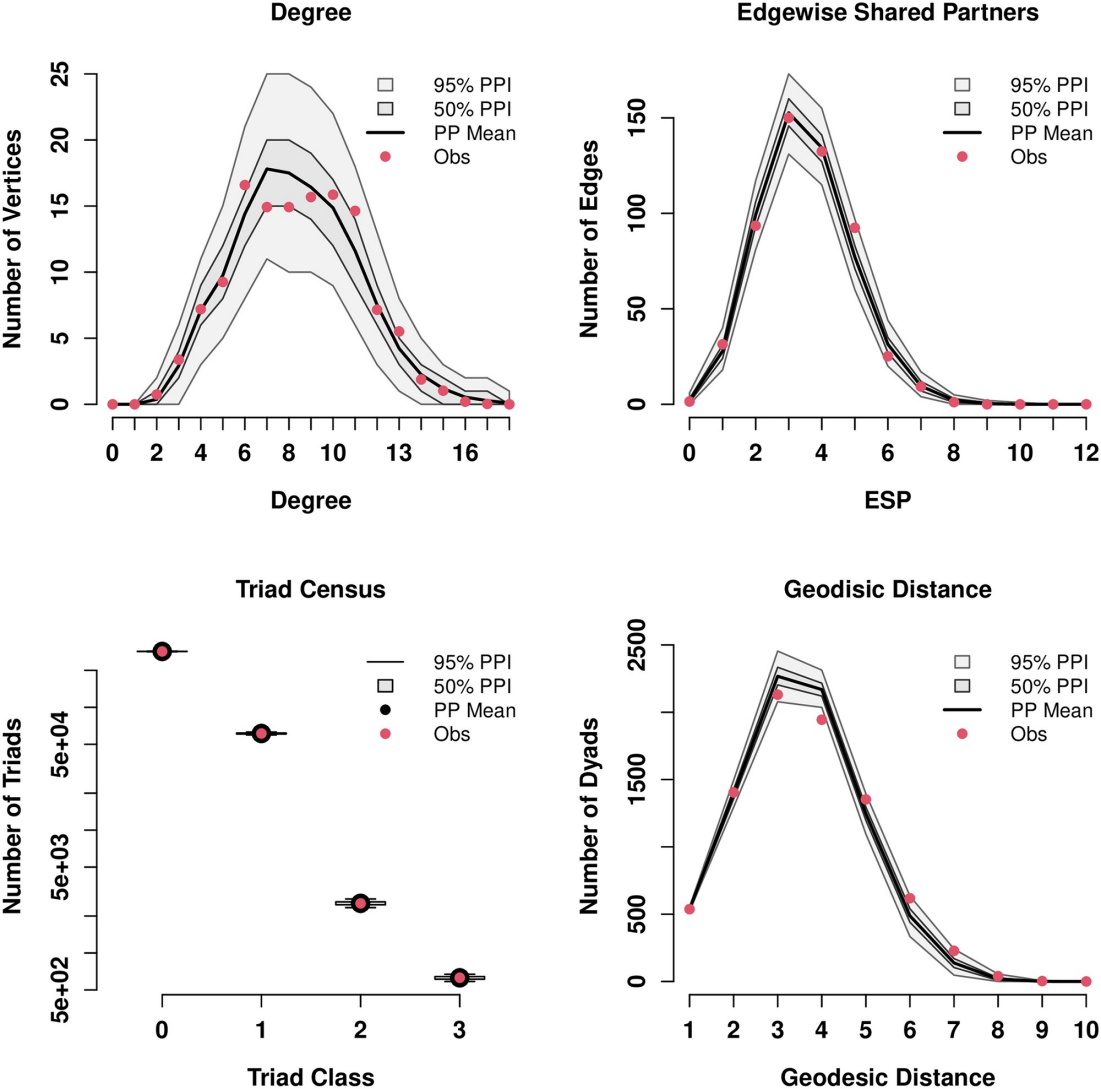
Model adequacy checks for the pooled lysozyme model; shaded areas/boxes show posterior predictive intervals, while red points indicate observed mean values. (Note that some intervals in the lower left panel are narrower than box line widths; all intervals are in fact vertical). The lysozyme model successfully recapitulates a range of structural features.

As a model adequacy check, we take 1000 draws from the posterior predictive distribution (based on the Laplace approximation), comparing the distribution of several standard structural properties (degree distribution, ESP distribution, geodesic distance distribution, and triad census) with the observed data means. The result is shown in Fig 5. As can be seen, the model is able to recapitulate all of the above features, indicating that it does a reasonable job of capturing the basic structural properties of the lysozyme networks.

#### 4.3.3 Reproducing structural variability

As noted above, one potential use for ERGM analysis of protein structures is to characterize variability, and to identify dimensions of structural variation that may be imperfectly constrained by available data. Here, we simulate draws from the fitted lysozyme model and examine their range of variation with respect to four basic graph-level indices (GLIs) found by [99] to distinguish protein structures. These are:

- *Transitivity* [108]—a standard measure of triadic closure in network analysis, transitivity reflects the compactness of a PSN in the sense that higher levels of transitivity are associated with the structures that are closely and uniformly packed.
- Standard deviation of *degree distribution*—a measure of the level of heterogeneity in local packing around chemical groups.
- Standard deviation of the *core number* [109]—an indicator of the degree of heterogeneity in structural cohesion, which distinguishes between highly organized structures and structures that combine rigidly and loosely bound regions.
- Standard deviation of *M-eccentricity*—the idea of *M-eccentricity* stems from *eccentricity* [110], and was introduced in the context of PSN analysis by [99]. The M-eccentricity of a vertex is the mean distance from that vertex to all other vertices; vertices with low M-eccentricity are more centrally located, while those with high M-eccentricity are peripheral to the graph structure. Thus the standard deviation of M-eccentricity distinguishes between uniformly globular structures and structures with deformations or other elongations.

**Fig 6:**
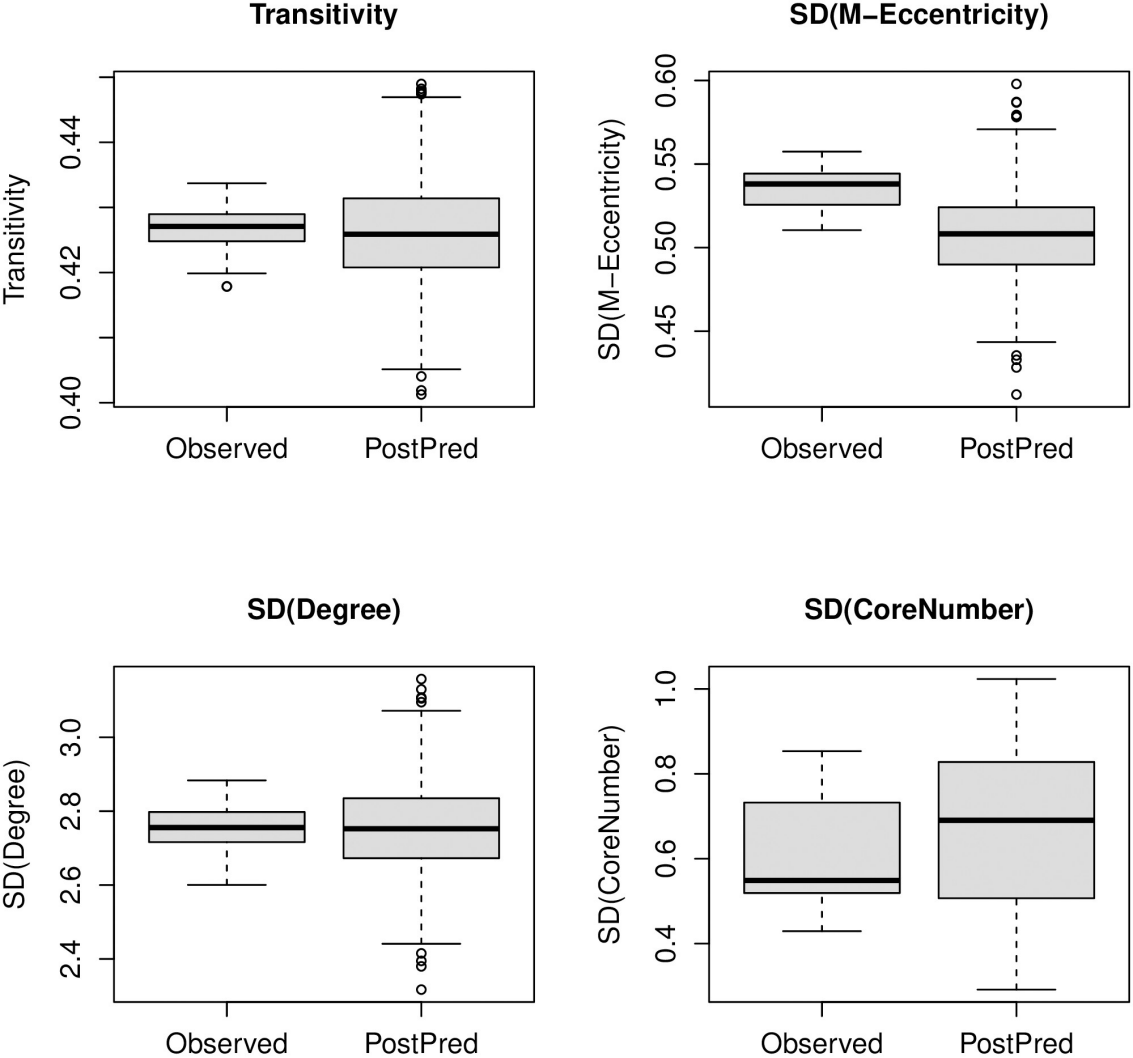
Variation in graph-level indices for lysozyme PSNs, observed versus posterior predictive distributions; simulated networks cover the observed GLI range, but also account for predictive uncertainty.

Fig 6 shows the distribution of the above GLI values for the observed lysozyme networks and for posterior predictive draws from the pooled ERGM; GLIs were calculated using the sna library for R [86]. For each of the GLI distributions, we can see that the posterior predictives cover the observed distributions, while being somewhat wider (reflecting posterior uncertainty). Such distributions have potential uses such as statistical comparison of protein families or variants from pooled crystallographic data, where accounting for uncertainty in the distribution of structural properties is an important consideration.

## 5 Discussion

Here, we briefly discuss several different issues related to the methods described here, particularly including missing data and model parameterization.

### 5.1 Missing data handling

Our technique depends upon the ability to compute $\bar{g}\left(y^{obs}\right)$; when some graphs contain missing (i.e., unobserved) edge variables, their statistics cannot in general be calculated, and neither therefore can $\bar{g}\left(y^{obs}\right)$. Although we do not treat extension to the incompletely observed case in detail, we here briefly sketch an approach to this problem. Our proposed scheme uses the EM algorithm [111], with a data augmentation scheme related to those of [33, 112]. Given our sample ***y***^**obs**^ = (*y*^1^, …, *y*^*m*^), let us divide the edge variables into observed ($y_{o}^{\text{obs}}=\left(y_{o}^{1},\ldots ,y_{o}^{m}\right)$) and missing ($y_{m}^{\text{obs}}=\left(y_{m}^{1},\ldots ,y_{m}^{m}\right)$) components. We begin with some initial guess at the parameter vector, *θ*^(0)^, and then proceed iteratively as follows. At the *i*th step: (1) draw $y_{m}^{\text{obs}}|y_{o}^{\text{obs}},\theta_{i-1}$ using $y_{m}^{j}\cup y_{o}^{j}∼ERGM\left(\theta_{i-1}\right)$; (2) compute $\bar{g}\left(y_{m}^{\text{obs}}\cup y_{o}^{\text{obs}}\right)$ (averaging over multiple imputation draws); and then (3) find $\theta_{i}|\bar{g}\left(y_{m}^{\text{obs}}\cup y_{o}^{\text{obs}}\right)$ via MLE or MAP as described elsewhere in the paper. This is repeated until convergence. This is a standard EM algorithm, with assumptions and solution properties equivalent to those of [112] (who use a nested MCMC strategy to perform Geyer-Thompson based maximum likelihood estimation in the single graph case).

We observe that the computational efficiency of this approach hinges on step (3), but can be undermined by step (1) in the case where missingness is extensive. In the extreme case in which all graphs contain significant amounts of missing data, this algorithm requires running MCMC over all graphs in the sample, and may not be dramatically more efficient than a conventionally pooled Handcock-Gile scheme. On the other hand, when missingness is confined to a small number of graphs, and/or when the number of edge variables to be simulated is small, then the savings from mean value inference in step (3) may still be considerable.

### 5.2 Pooling versus other approaches

As noted in the introduction, pooling is only one of several approaches for modeling graph sets (lying at one end of continuum that passes from pooling to hierarchical and mixed models, and thence to independent estimation). Pooling is a useful strategy when we either have reason to believe that our sample was drawn independently from a common generating process, or when we wish to use an approximation of this type. For the pooling procedure described here, we note that we are by assumption working with networks of the same size, sharing the same covariates; many common cases where heterogeneity is a serious concern (e.g., classrooms of differing size and composition) are thus beyond the scope of our technique. For graph sets on equivalent vertices, it may be helpful to assess pooling success using analyses like those in Section 4.3.3. If variation in observed network properties greatly exceeds those of the simulated models, then this may suggest that the sample arose from a mixture of generative processes; in this case, latent ERGM mixture models like those of [113] may be helpful in detecting and fitting parameters for the underlying mixture components. Alternately, such models may simply require elaboration. For instance, [114] shows that cross-graph variation in density and/or reciprocity can be captured in pooled models using an appropriate choice of ERGM terms. Excess cross-graph variation does not therefore imply that a pooled model cannot be successful, though it suggests that closer inspection may be in order. As in other matters, adequacy of the pooled approximation depends on the purposes of the associated analysis.

### 5.3 Pooling and model complexity

Models for complex networks can themselves become quite complex, raising practical, statistical, computational, and interpretational issues. How many terms are “too many,” and should one err on the side of simple models (which are easier to understand and work with, but that may omit important confounders or mechanisms) or higher-dimensional models (which may account for more drivers of network structure, at the expense of computational cost, interpretability, and overfitting risk)? This debate (analogous to the “emulative” versus “intellective” modeling discussion in the agent-based literature [115]) involves inherent tradeoffs between respective modeling strategies, and is in our view ill-posed: models at many different levels of complexity can be useful, and the objective should be to match the capacities and requirements of a model to the uses to which it will be put.

Pooling enters into this discussion in two respects. First, as noted above, models for populations of graphs may require greater complexity to account for cross-graph variation than models for a single graph. For instance, excess variation in density may be accounted for by a change of reference, together with the inclusion of terms for both log edge count and log null count (instead of the usual edge count statistic) [114]; this may account for greater structural variability, but at some cost to parsimony and ease of interpretation. Second, pooled models allow for considerably greater statistical power than single-graph models, particularly where sample sizes are large. This increase in power both makes it possible to reliably fit higher-dimensional models, and to detect very small deviations from the no-effect null hypothesis (i.e., one can very easily reject the hypothesis that a hypothesized term has no effect, even if the parameter has little impact on model behavior). In our view, both considerations strengthen the importance of substantively guided model selection based on a clear sense of modeling objectives. On the one hand, positing a model that will capture all aspects of a large graph set is rarely realistic, and one must thus choose what one will—and will not—seek to capture. And on the other hand, in the large-data regime one cannot count on statistical power or null hypothesis tests to tell one what one should or should not include in a practical model. Particularly as the methods presented here make it computationally feasible to fit models to very large graph sets, the analyst (at least in the equivalent vertex setting) is more often able to shift from asking what models they can fit to what models they *should* fit. We suggest that this shift motivates further work on substantive adequacy checking for network models in particular settings, particularly including efforts to link predicted network structure with other, non-network data or outcomes. For instance, Friedkin’s ([116]) experimental studies of influence in task groups reliably link equilibrium attitudes to influence networks in ways that suggest that attitudinal configurations could be predicted in part from network models even where the networks themselves cannot be observed; adequacy for this task may be rather different than what is prioritized by conventional metrics. As our ability to efficiently fit network models improves, pursuing such questions becomes an important priority.

## 6 Conclusion

We have presented a highly scalable approach for modeling multiple network observations with ERGMs, under both frequentist and Bayesian paradigms, utilizing basic exponential family properties to perform pooling and/or Bayesian updating entirely within the mean value space. This allows us to perform inference on arbitrary numbers of graphs at no additional cost, and to perform Bayesian inference at the same cost as maximum likelihood estimation. Moreover, by mapping the inferential problem to a problem involving a single network, it is possible to perform both pooled and Bayesian inference with standard software packages designed for single-network applications, without resorting to techniques like graph aggregation with structural zeros that add complexity and computational cost. Simulation experiments suggest that the frequentist properties of the pooling procedure are quite good (with minimal bias and good calibration with even small sample sizes), and conjugate-prior MAP inference yields well-behaved interpolation between prior parameters and the MLE. Conjugate-prior MAP estimates with a simple default prior were also found to have good frequentist properties for a range of diffuse prior weights, suggesting its value as a simple tool for regularized inference (with the most important use case being settings where the MLE does not exist due to the convex hull problem). Although this work focused on a specific choice of default prior that is analogous to a zero vector in the natural parameter space (with the exception of the edge parameter which is corrected for prior density)—a natural analog to the zero-centered priors used in existing strategies for Bayesian ERGM inference—the fact that the conjugate prior is specified in the mean value space (i.e., the space of graph statistics) makes it particularly easy to specify informative alternatives based on e.g., prior data sets.

We demonstrated the applicability of our inferential scheme with two applications, specifically to brain functional connectivity networks and to protein structure networks. In both cases, the ability to quickly and easily pool network data without additional computational cost, and to easily use either Bayesian or frequentist inference, facilitates analysis. We also show how the regularization offered by the use of prior structure makes it possible to include theoretically interesting mixing terms that (because their statistics lie on the convex hull) are problematic under MLE, and how prior substantive information (here, simple empirical models of the properties of monomeric proteins) can be used to create reasonable prior specifications even without existing network data.

The results shown here were produced using the MCMC MLE estimation strategy used by the ergm package, but the idea can be easily adapted to any other ERGM estimation scheme based on fitting to the sufficient statistics (e.g., contrastive divergence, stochastic approximation, the log partition function scheme of [117], or other forms of gradient descent). It is not compatible with approximate likelihood methods such as maximum pseudo-likelihood estimation (MPLE) that operate directly on edge variables, although we observe that it is still possible to initialize estimation by MPLE on a single graph from the set and then proceed with methods based on statistics (as was in fact done here), or otherwise use methods such as contrastive divergence that are similar in speed and accuracy. We do note that one side effect of the high level of statistical precision obtainable from pooled network models is that *de facto* accuracy eventually begins to depend more on numerical error than statistical uncertainty. While we find that calibration remains good for the range of data sizes considered in our simulation study, precise inference for very large collections of networks may require greater attention to numerical stability than is necessary for conventional ERGM inference. Efficient high-precision algorithms for pooled models in the large-*m* regime would seem to be an important problem for future work.

## Supporting information

S1 Appendix (PDF)

S1 Fig Model adequacy checks for the pooled brain connectivity network ML-estimated model. Shaded areas/boxes show simulation intervals, while red points indicate observed mean values. (Note that some intervals in the lower left panel are narrower than box line widths; all intervals are in fact vertical).
(PDF)

S2 Fig Model adequacy checks for the pooled brain connectivity network MAP-estimated model. Shaded areas/boxes show simulation intervals, while red points indicate observed mean values. (Note that some intervals in the lower left panel are narrower than box line widths; all intervals are in fact vertical).
(PDF)

S3 Fig Model adequacy checks for the pooled brain connectivity network regularized ML-estimated model. Shaded areas/boxes show simulation intervals, while red points indicate observed mean values. (Note that some intervals in the lower left panel are narrower than box line widths; all intervals are in fact vertical).
(PDF)
