# Supplementary material for: Highly scalable maximum likelihood and conjugate Bayesian inference for ERGMs on graph sets with equivalent vertices
Source: PLoS One. 2022 Aug 26;17(8):e0273039. doi: 10.1371/journal.pone.0273039 (PMC9417041; doi:10.1371/journal.pone.0273039)
Supplement: S1 Appendix — (PDF) [file pone.0273039.s001.pdf]

Appendix A

For convenience, we here provide the definitions of a number of common families of ERGM terms used in this paper.

`Edges`

$$t_e(y) = \sum_{i<i} y_{ij}$$

This term counts the number of edges within the graph; since the graphs used here are all undirected, we show the undirected version. The edge term acts as a *de facto* intercept for the model, setting the baseline tie probability, and is central in fixing the expected density of the network.

`Geometrically weighted edgewise shared partners (GWESP)`

$$t_g(y, \phi) = e^{\phi} \sum_{k=1}^{n-2} \left\{1 - (1 - e^{-\phi})^k\right\} EP_k(y)$$

where $EP_k(y)$ is the number of connected pairs that have exactly k common neighbors, which is a measure of local clustering in network research. The decay parameter ϕ controls the relative contribution of $EP_k(y)$ to the GWESP statistic. A positive, large coefficient on GWESP term tends to concentrate more probability mass on the graphs with more local clustering.

Nodematch

Given a categorical covariate x defined for each node in the graph, a nodematch term counts the total number of edges between nodes sharing same value on x,

$$t_H(y;x) = \sum_{i<j} y_{ij} \mathbb{1}_{\{x_i = x_j\}}$$

Nodemix

Given a categorical covariate x defined for each node in the graph, a nodemix term counts the total number of edges between nodes of level k and level l on x,

$$t_M(y;x) = \sum_{i<j} y_{ij} \mathbb{1}_{\{(x_i,x_j)=(k,l) \text{ or } (x_i,x_j)=(l,k)\}}.$$

The parameter associated with a k, l nodemix statistic inhibits or enhances the rate at which nodes with respective values k and l on x interact with one another; negative values hinder interaction, while positive values enhance it.

Nodecov

Given a covariate x defined for each node in the graph, a nodecov term is defined as follows:

$$t_{NC}(y;x) = \sum_{i<j} y_{ij}(x_i + x_j)$$

This statistic captures the degree of association between nodes' values of x and their net tendency to send or receive ties.

Edgecov

Given a covariate x defined for each pair of nodes in the graph, an edgecov term is defined as follows:

$$t_{EC}(y;x) = \sum_{i<j} y_{ij} x_{ij}$$

This statistic can be viewed as the product-moment of the elements of the adjacency matrix with the elements of x, and as such captures the degree of association between dyads' values on x and the presence or absence of edges.

`2-stars`

$$t_{2S}(y) = \sum_{i<j<k} y_{ik}y_{jk}$$

This statistic counts the number of 2-star configurations within the graph (i.e., a subgraph containing node with two simultaneous partners). The associated parameter can be viewed as indicating the effect of each of a node's current edges on the conditional log odds of a specific other edge involving that node being present; when negative, in particular, it can be viewed as a first-order model for hindrance (in which each existing edge makes it more difficult to acquire new edges).

`Triangles`

$$t_T(y) = \sum_{i<j<k} y_{ik}y_{jk}y_{ij}$$

This statistic counts the number of triangles with in the graph (i.e., complete subgraphs of order three). The associated parameter can be viewed as indicating the effect of each of a node pair's shared partners on the conditional log odds of being tied. Although prone to producing runaway clique formation when associated with a positive parameter (the famous "density explosion" path to degeneracy), negative triangle parameters can serve to inhibit the formation of large cliques (since every clique of order k contains on the order of k^3 triangles) and can serve as a model for packing constraints.

`graphletCount`

This statistic counts the number of times that a specific graphlet appears in a network, and can be added to ERGM via `graphletCount` command (see [95] for more details about its definition and implementation). Specifically, we use the `graphletCount(1)` statistic in some of our models. Graphlet 1 (in the [95] nomenclature) is an open 2-path: i.e., a configuration such that, for vertices i, j, k, there is an (i, j, k) 2-path with no i, k edge. Formally,

$$t_{G_1}(y) = \sum_{i<j<k} y_{ij}y_{jk}(1 - y_{ij}).$$

(Compare with the 2-star and triangle statistics, which are respectively indifferent to or require closure.)

The open 2-path count is closely related to the *null shared partner* statistics, of which the kth (NSP(k)) is the number of nulls (non-adjacent vertex pairs) having exactly k shared partners. Clearly, each NSP(k) adds k open 2-paths to the graph. [42, 88] use a geometrically weighted NSP statistic (compare with GWESP, above) to model bridging in brain functional networks. Here, we use the open 2-path count instead of GWNSP, as we found the former to be more stable than the latter when fitting models using the current generation of software tools, on the specific data set examined here.
