# Supplementary figures and images for: Highly scalable maximum likelihood and conjugate Bayesian inference for ERGMs on graph sets with equivalent vertices
Source: PLoS One. 2022 Aug 26;17(8):e0273039. doi: 10.1371/journal.pone.0273039 (PMC9417041; doi:10.1371/journal.pone.0273039)

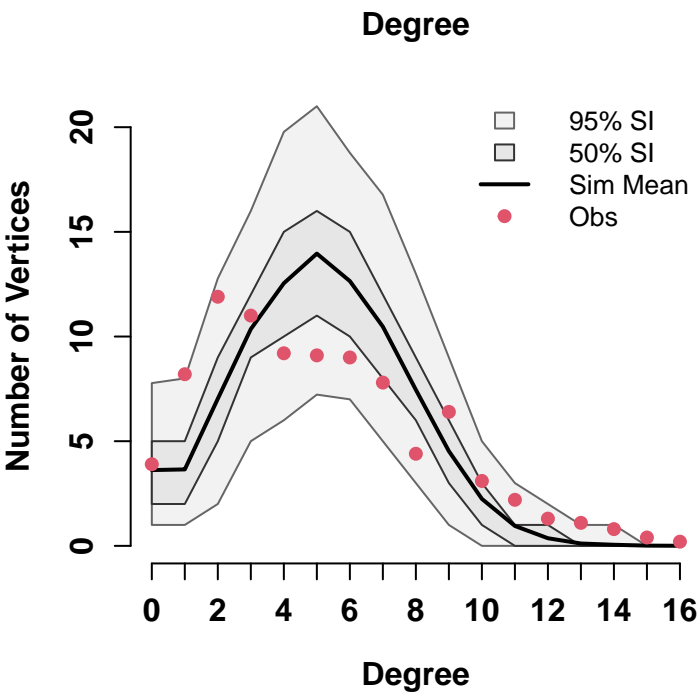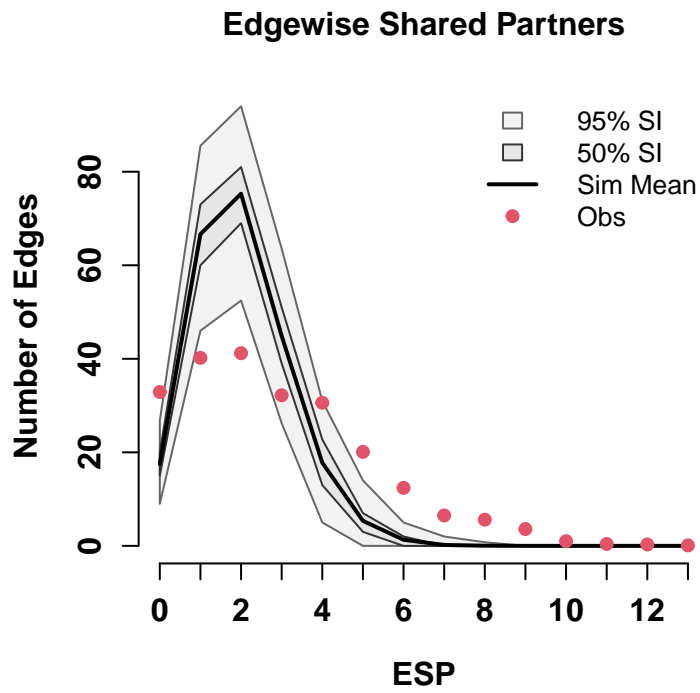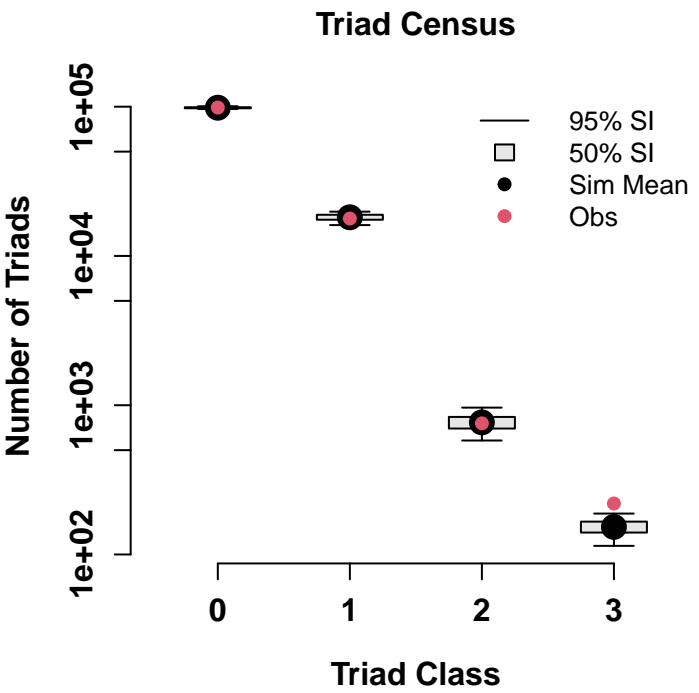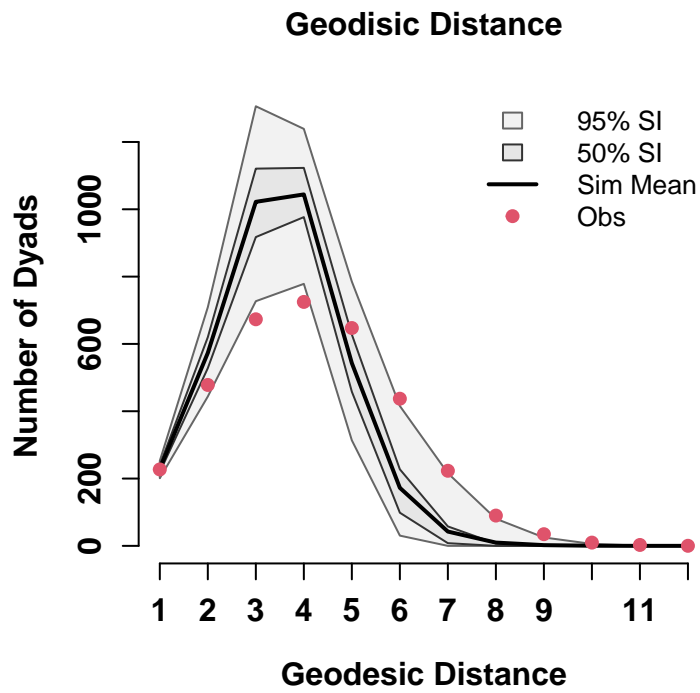

Supplement: S1 Fig — Shaded areas/boxes show simulation intervals, while red points indicate observed mean values. (Note that some intervals in the lower left panel are narrower than box line widths; all intervals are in fact vertical). (PDF) [file pone.0273039.s002.pdf]

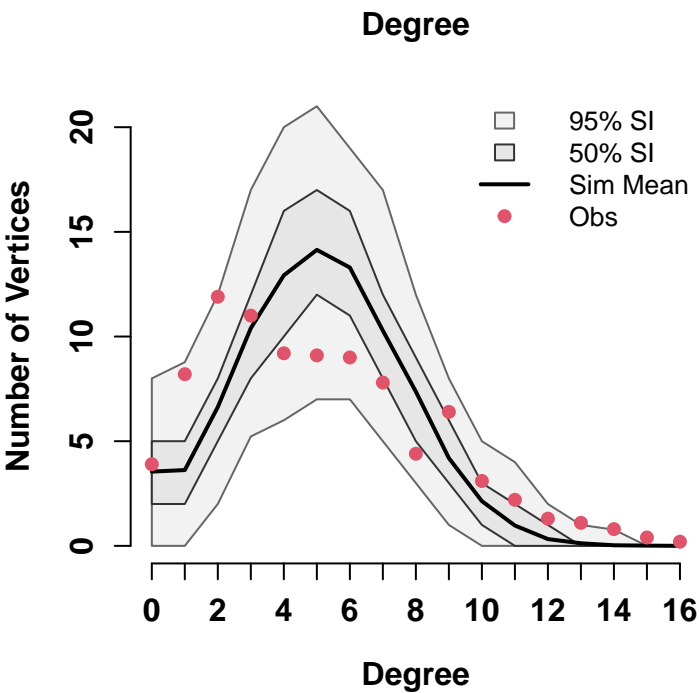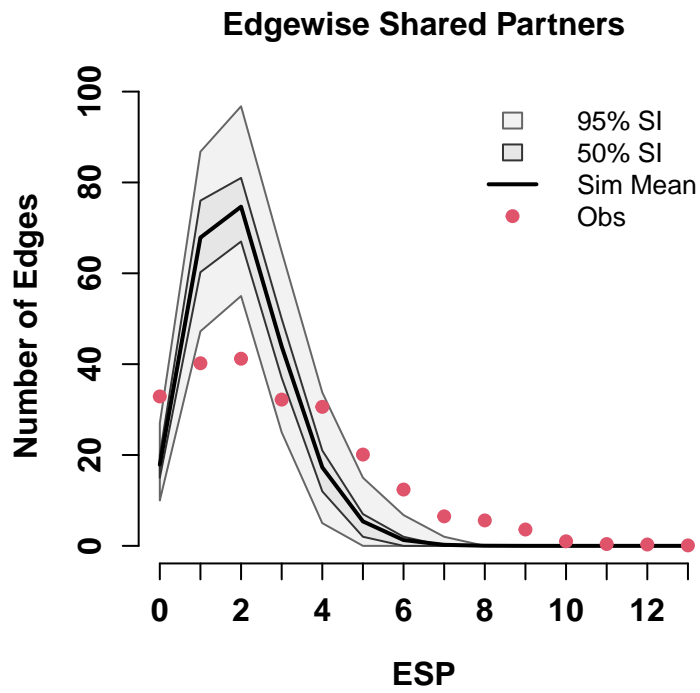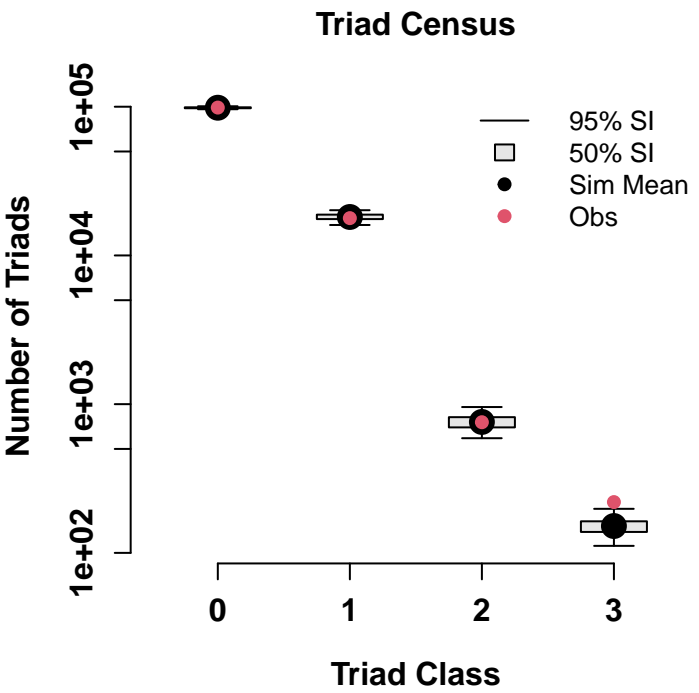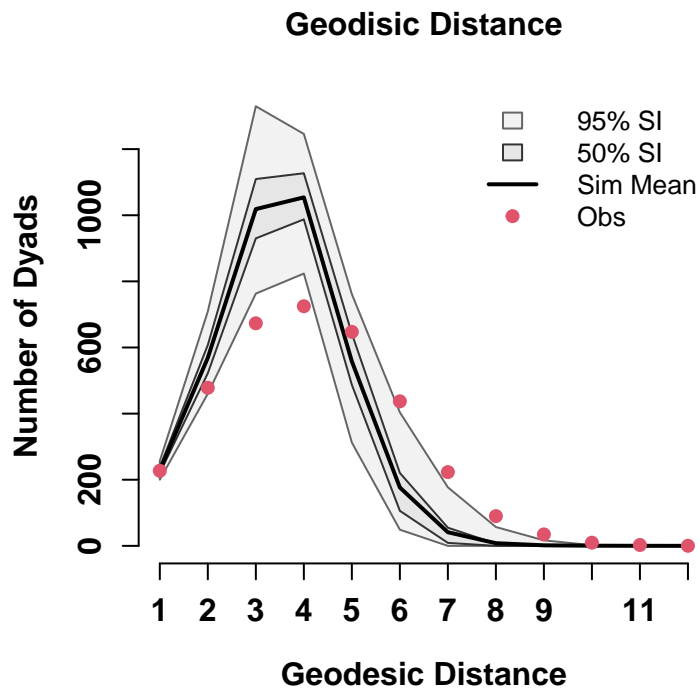

Supplement: S2 Fig — Shaded areas/boxes show simulation intervals, while red points indicate observed mean values. (Note that some intervals in the lower left panel are narrower than box line widths; all intervals are in fact vertical). (PDF) [file pone.0273039.s003.pdf]

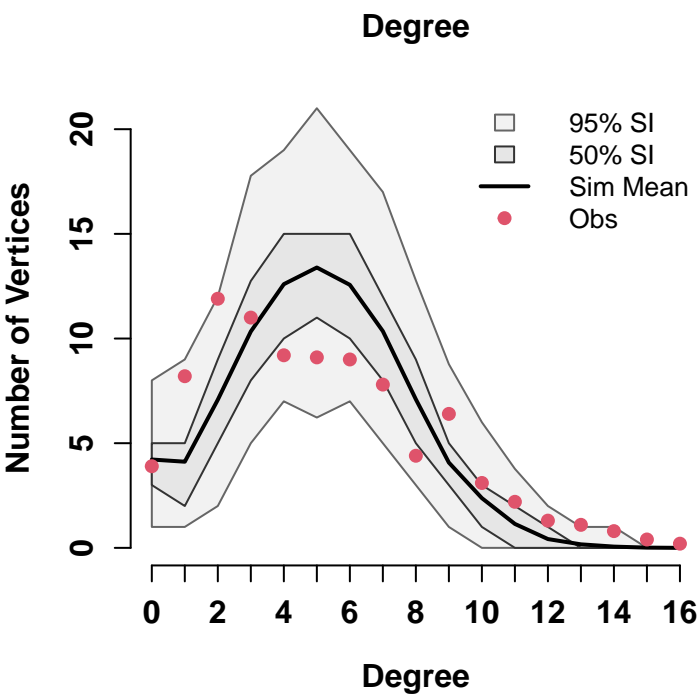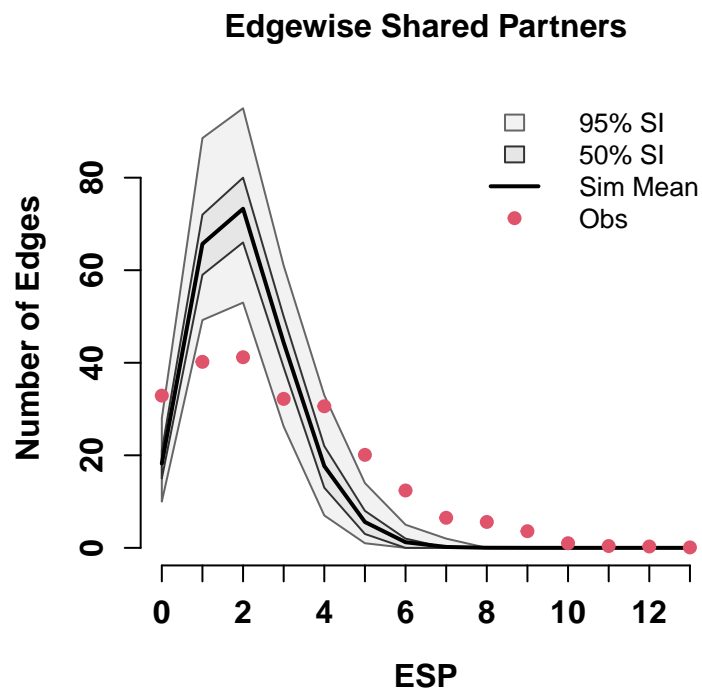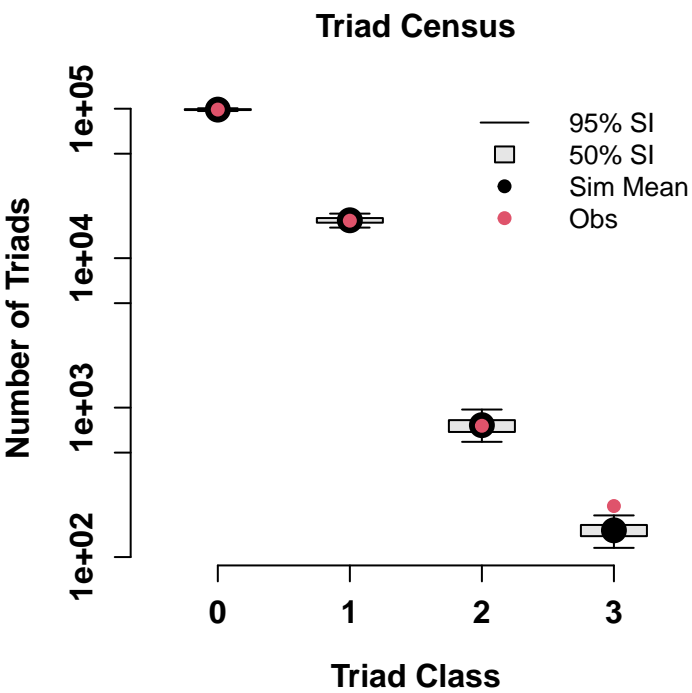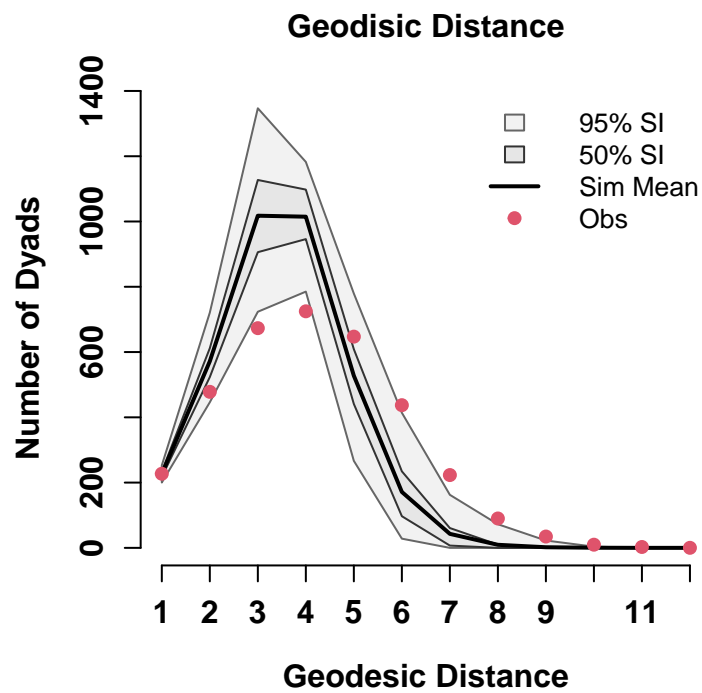

Supplement: S3 Fig — Shaded areas/boxes show simulation intervals, while red points indicate observed mean values. (Note that some intervals in the lower left panel are narrower than box line widths; all intervals are in fact vertical). (PDF) [file pone.0273039.s004.pdf]
